# Methyl-CpG-binding domain 9 (MBD9) is required for H2A.Z incorporation into chromatin at a subset of H2A.Z-enriched regions in the Arabidopsis genome

**DOI:** 10.1371/journal.pgen.1008326

**Published:** 2019-08-05

**Authors:** Paja Sijacic, Dylan H. Holder, Marko Bajic, Roger B. Deal

**Affiliations:** 1 Department of Biology, Emory University, Atlanta, GA, United States of America; 2 Graduate Program in Genetics and Molecular Biology, Emory University, Atlanta, GA, United States of America; Swedish University of Agricultural Sciences (SLU), SWEDEN

## Abstract

The SWR1 chromatin remodeling complex, which deposits the histone variant H2A.Z into nucleosomes, has been well characterized in yeast and animals, but its composition in plants has remained uncertain. We used the conserved SWR1 subunit ACTIN RELATED PROTEIN 6 (ARP6) as bait in tandem affinity purification experiments to isolate associated proteins from *Arabidopsis thaliana*. We identified all 11 subunits found in yeast SWR1 and the homologous mammalian SRCAP complexes, demonstrating that this complex is conserved in plants. We also identified several additional proteins not previously associated with SWR1, including Methyl-CpG-BINDING DOMAIN 9 (MBD9) and three members of the Alfin1-like protein family, all of which have been shown to bind modified histone tails. Since *mbd9* mutant plants were phenotypically similar to *arp6* mutants, we explored a potential role for MBD9 in H2A.Z deposition. We found that MBD9 is required for proper H2A.Z incorporation at thousands of discrete sites, which represent a subset of the genomic regions normally enriched with H2A.Z. We also discovered that MBD9 preferentially interacts with acetylated histone H4 peptides, as well as those carrying mono- or dimethylated H3 lysine 4, or dimethylated H3 arginine 2 or 8. Considering that MBD9-dependent H2A.Z sites show a distinct histone modification profile, we propose that MBD9 recognizes particular nucleosome modifications via its PHD- and Bromo-domains and thereby guides SWR1 to these sites for H2A.Z deposition. Our data establish the SWR1 complex as being conserved across eukaryotes and suggest that MBD9 may be involved in targeting the complex to specific genomic sites through nucleosomal interactions. The finding that MBD9 does not appear to be a core subunit of the Arabidopsis SWR1 complex, along with the synergistic phenotype of *arp6;mbd9* double mutants, suggests that MBD9 also has important roles beyond H2A.Z deposition.

## Author summary

The histone H2A variant, H2A.Z, is found in all known eukaryotes and plays important roles in transcriptional regulation. H2A.Z is selectively incorporated into nucleosomes within many genes by the activity of a conserved ATP-dependent chromatin remodeling complex in yeast, insects, and mammals. Whether this complex exists in the same form in plants, and how the complex is targeted to specific genomic locations have remained open questions. In this study we demonstrate that plants do indeed utilize a complex analogous to those of fungi and animals to deposit H2A.Z, and we also identify several new proteins that interact with this complex. We found that one such interactor, Methyl-CpG-BINDING DOMAIN 9 (MBD9), is required for H2A.Z incorporation at thousands of genomic sites that share a distinct histone modification profile. The histone binding properties of MBD9 suggest that it may guide H2A.Z deposition to specific sites by interacting with modified nucleosomes and with the H2A.Z deposition complex. We hypothesize that this represents a general paradigm for the targeting of H2A.Z to specific sites.

## Introduction

Nucleosomes, the fundamental units of chromatin that consist of ~147 bp of DNA wrapped around a histone octamer, efficiently condense large eukaryotic DNA molecules inside the nucleus. At the same time, nucleosomes present a physical barrier that restricts the access of DNA-binding proteins to regulatory sequences. This physical constraint imposed by nucleosomes on DNA can be modulated to expose or occlude regulatory DNA sequences, and is thereby used as a mechanism to control processes such as transcription that rely on sequence-specific DNA binding proteins. Thus, enzymatic complexes that can remodel chromatin structure by manipulating the position and/or composition of nucleosomes are essential for proper transcriptional regulation and the execution of key developmental programs.

All chromatin-remodeling complexes (CRCs) contain a DNA-dependent ATPase catalytic subunit that belongs to the SNF2 family of DNA helicases, along with one or more associated subunits [1, 2]. There are four major subfamilies of CRCs: SWI/SNF, INO80, ISWI, and CHD, all of which use the energy of ATP to either slide, evict, or displace nucleosomes, or to replace the canonical histones within nucleosomes with histone variants. One member of the INO80 CRC subfamily is the SWI2/SNF2-related 1 (SWR1) chromatin remodeler, a multisubunit protein complex required for incorporation of the H2A variant, H2A.Z, into chromatin [3, 4]. H2A.Z is a highly conserved histone variant found in all eukaryotes that plays important roles in regulating a variety of cellular processes, including transcriptional activation and repression, maintenance of genome stability and DNA repair, telomere silencing, and prevention of heterochromatin spreading [5–12]. Although loss of H2A.Z is not lethal in yeast [2, 13], H2A.Z is essential for viability in other organisms such as Tetrahymena [14], Drosophila [15, 16], and mice [17]. Interestingly, H2A.Z-deficient plants are viable but display many developmental abnormalities such as early flowering, reduced plant size, altered leaf morphology, and reduced fertility [18–20].

The SWR1 complex that mediates H2A.Z incorporation into chromatin was first described in yeast and is composed of 13 subunits, including Swr1, the catalytic and scaffolding subunit of the complex [3, 4, 21, 22]. In mammals, the functional and structural homolog of yeast SWR1 complex is the SRCAP (SNF2-related CREB-binding protein activator protein) complex. This complex is composed of 11 of the same subunits found in yeast SWR1 and is likewise able to exchange H2A/H2B dimers for H2A.Z/H2B dimers in nucleosomes [23–26]. Intriguingly, higher eukaryotes possess an additional multi-subunit complex, 60 kDa Tat-interactive protein (TIP60), that has histone acetyltransferase (HAT) activity and can also mediate the deposition of H2A.Z into nucleosomes [27–30]. Furthermore, the yeast Nucleosome Acetyltransferase of histone 4 (NuA4) complex, which is structurally related to SWR1 through the sharing of four subunits [31–35], was shown to regulate the incorporation of H2A.Z into chromatin cooperatively with the SWR1 complex [34].

Many homologs of yeast SWR1 and animal SRCAP complex subunits have been identified in *Arabidopsis thaliana* including ACTIN RELATED PROTEIN 6 (ARP6), SWR1 COMPLEX SUBUNIT 2 (SWC2), SWC6, PHOTOPERIOD-INDEPENDENT EARLY FLOWERING 1 (PIE1), and three H2A.Z paralogs: HTA8, HTA9, and HTA11. Numerous genetic and biochemical experiments suggest that the SWR1 complex is conserved in Arabidopsis. For example, it has been recently shown that Arabidopsis SWC4 protein directly interacts with SWC6 and YAF9a, two known components of the SWR1 complex [36]. Additionally, protein interaction experiments have demonstrated that PIE1 interacts directly with ARP6, SWC6, HTA8, HTA9, and HTA11 [18, 20, 37, 38], suggesting that PIE1 may serve as the catalytic and scaffolding subunit of an Arabidopsis SWR1-like complex. Furthermore, functional characterizations of *PIE1*, *ARP6* and *SWC6* have revealed that mutations in these genes have similar pleiotropic effects on Arabidopsis development, including a loss of apical dominance, curly and serrated rosette leaves, early flowering due to reduced expression of *FLOWERING LOCUS C* (*FLC*), altered petal number, and reduced fertility [18, 37–43]. Interestingly, genetic experiments revealed that the *pie1* null phenotypes are more severe than those of *arp6*, *swc6*, *hta9;hta11*, or *hta8;hta9;hta11* (*h2a*.*z* near-null) plants [18, 19, 37–43]. The more dramatic phenotypes in *pie1* plants suggest that PIE1 has additional functions outside of H2A.Z deposition by SWR1, as previously proposed [5, 6, 18, 19]. A recent report also showed that mutant plants null for *pie1* and *h2az* exhibited early developmental arrest, dying shortly after germination [19], further supporting the notion that PIE1 has H2A.Z-independent functions in Arabidopsis. On the other hand, genetic analyses of *pie1;swc6* double mutant plants revealed that they had a phenotype indistinguishable from *pie1* single mutants [38], and *arp6;swc6* plants displayed the same defects as either *arp6* or *swc6* single mutant plants [18, 37]. These results further support the idea that PIE1, ARP6, and SWC6 act in the same genetic pathway and/or are the components of the same protein complex, but that PIE1 has additional functions.

Despite the strong genetic and biochemical evidence that Arabidopsis contains many conserved subunits homologous to the components of the yeast SWR1 complex and mammalian SRCAP, the plant SWR1 complex has not been successfully isolated and characterized. Recently, Bieluszewski and colleagues used Arabidopsis SWC4 and ARP4 proteins, two subunits shared between yeast SWR1 and NuA4, as well as mammalian SRCAP and TIP60 complexes, as baits to affinity purify their interacting partners from Arabidopsis cell suspension cultures [44]. These studies identified most of the subunits normally found in the SWR1 and NuA4 complexes, but it was not possible to determine whether this collection of proteins represented a single large complex or multiple complexes. Overall, it is not yet clear whether plants possess separate SWR1 and NuA4 complexes, SWR1 and TIP60-like complexes, or all three complexes [44].

The main goal of our study was to purify the Arabidopsis SWR1 complex in order to identify all of its components. To achieve this, we used the ARP6 protein, a subunit unique to SWR1 in other organisms, as bait in Tandem Affinity Purification (TAP) experiments. We performed three independent TAP experiments to isolate and identify ARP6-associated proteins. We identified all 11 conserved subunits found in yeast SWR1 and mammalian SRCAP complexes, demonstrating that Arabidopsis contains a bona-fide functional and structural homolog of these complexes. In addition, we identified several unexpected proteins that associated with ARP6, including the plant homeodomain (PHD)- and Bromo domain-containing protein Methyl CpG-BINDING DOMAIN 9 (MBD9), and the PHD domain-containing proteins ALFIN-LIKE 5 (AL5), AL6, and AL7. Association of these proteins with the SWR1 complex is consistent with the results of a recent related study that used ARP6-MYC or -FLAG tag epitope purification followed by mass spectrometry to identify ARP6-interacting proteins (Potok et al. 2019, bioRxiv 10.1101/657296). Genetic analyses revealed that *mbd9* mutants showed phenotypic similarities to *arp6* mutants, so we further explored a possible role for MBD9 in regulating H2A.Z incorporation into chromatin. We found that MBD9 is required for H2A.Z incorporation at a subset of the sites that normally harbor H2A.Z nucleosomes, and that these MBD9-dependent H2A.Z sites have distinct chromatin features. Furthermore, MBD9 strongly interacted with acetylated histone H4 peptides, H3 peptides mono- or dimethylated at lysine 4, as well as H3 dimethylated at arginines 2 or 8. We also found that MBD9 is not a core subunit of the Arabidopsis SWR1 complex and that double mutant *arp6;mbd9* plants exhibited much more severe phenotypes than single *arp6* or *mbd9* mutants. These results collectively suggest that MBD9 targets the SWR1 complex to a subset of genomic loci but also has important functions beyond H2A.Z deposition.

## Results

### ARP6 transgenes tagged with a Tandem Affinity Purification tag rescue the *arp6-1* phenotype

**Fig 1:**
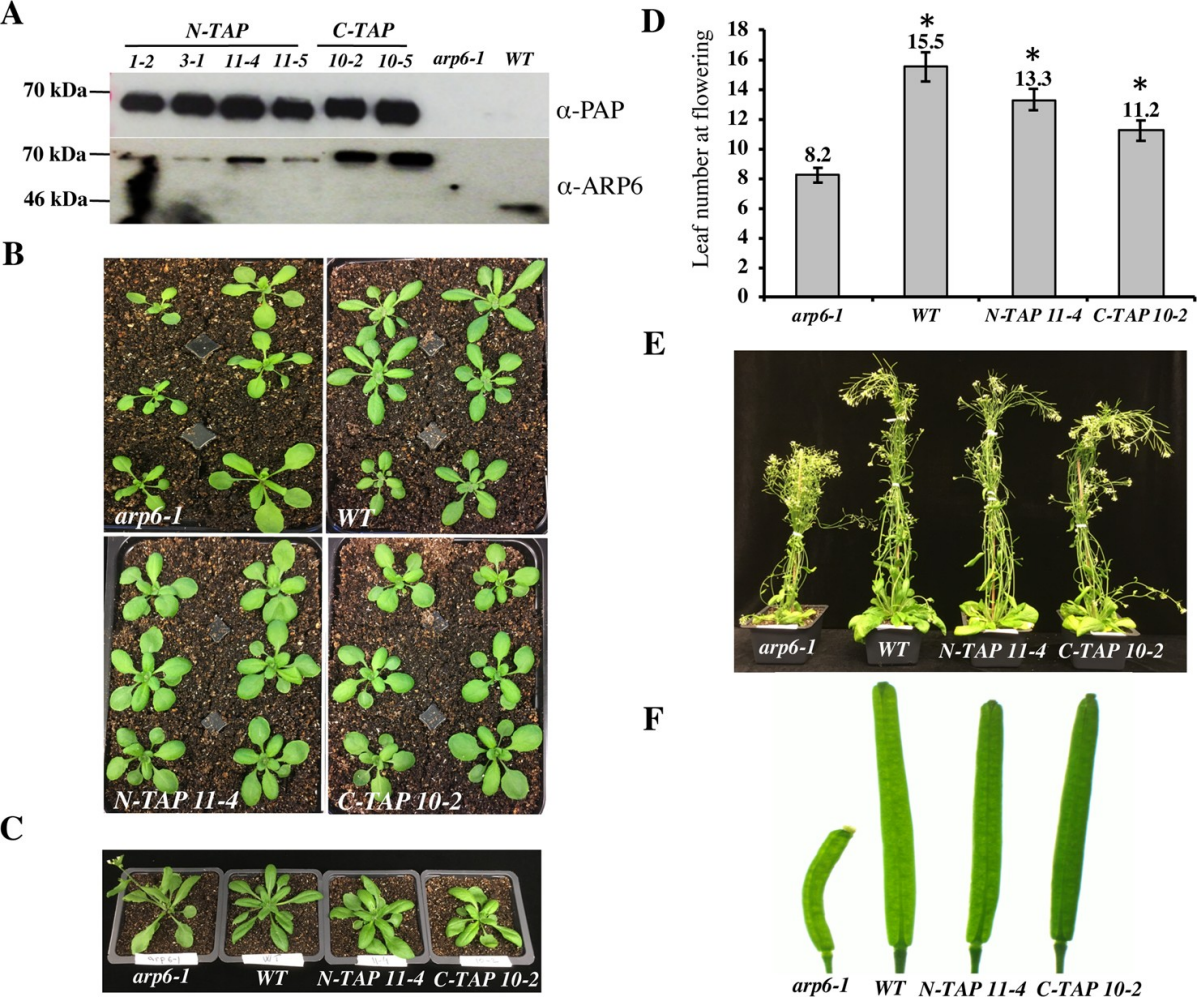
*N-TAP-ARP6* and *C-TAP-ARP6* transgenes rescue the *arp6-1* phenotype. (A- upper blot) T_2_ plants homozygous for *N-TAP-* or *C-TAP-ARP6* transgenes express a fusion protein with the expected size of 67.5 kDa. The fusion protein is specifically detected only in transgenic plants and not in *arp6-1* or WT plants using a peroxidase anti-peroxidase (PAP) soluble complex, which binds the protein A moiety of the TAP tag. (A- lower blot) The same protein extracts as in the upper blot were probed with a monoclonal ARP6 antibody. ARP6 presence is specifically detected in all transgenic plants as a 67.5 kDa fusion protein band compared to the 44 kDa ARP6 band in WT plants, and is absent in *arp6-1* mutant plants. The ARP6 antibody reacts less strongly with *N-TAP-ARP6*, most likely because the antibody recognizes the N-terminal region, which is adjoined to the TAP tag in this fusion. (B) Transgenic plants look more similar to WT plants than *arp6-1* plants, with more compacted, non-serrated rosette leaves. (C) Early flowering phenotype is rescued in transgenic plants when compared to the *arp6-1*. (D) The average number of rosette leaves of *N-TAP-ARP6* and *C-TAP-ARP6* transgenic plants at flowering is significantly higher than in *arp6-1* (n = 6 for WT and *arp6-1*, and n = 12 for *N-TAP 11–4* and *C-TAP 10–2*). Asterisks indicate significant differences from *arp6-1* plants with p<0.001, calculated using unpaired t-tests. (E) The loss of apical dominance defects of *arp6-1* plants are rescued in *N-TAP-ARP6* and *C-TAP-ARP6* transgenic plants. (F) The fertility defects of *arp6-1* plants are rescued in *N-TAP 11–4* and *C-TAP 10–2* transgenic plants.

To isolate the Arabidopsis SWR1 complex, we decided to use ARP6 protein as bait in Tandem Affinity Purification (TAP) experiments because ARP6 is exclusively found in the SWR1 complex in other organisms and is not shared by any other known CRCs [4, 18, 34]. For our purification experiments we used a GS^rhino^ TAP-tag, which consists of two protein G domains, a tandem repeat of rhinovirus 3C protease cleavage site, and the streptavidin-binding peptide. This tag has been successfully used to purify several plant nuclear complexes, including SWI/SNF type CRCs [45]. Furthermore, the use of this tag provides high yield of purified proteins and specificity of purification. In addition, a list of 760 proteins that nonspecifically bind to this tag or the associated purification beads has been assembled from data on 543 GS-based TAP experiments [45]. We fused the GS^rhino^ TAP-tag to either the N-terminal end (*N-TAP-ARP6*) or the C-terminal end (*C-TAP-ARP6*) of the genomic ARP6 coding sequence, containing the endogenous *ARP6* promoter, and introduced the constructs into *arp6-1* mutant plants to test for the ability of each transgene to complement a null *arp6* allele. Using western blotting, we first detected the presence of the 67 kDa ARP6-TAP-tag fusion protein specifically in plants homozygous for the transgene and not in *arp6-1* or wild-type (WT) plants (Fig 1A). Next, we assessed the ability of the transgenes to rescue the morphological defects of *arp6-1* plants. When grown in parallel, the transgenic plants appeared almost indistinguishable from WT plants, with more compacted, non-serrated rosette leaves as compared to *arp6-1* mutants (Fig 1B). The *N-TAP-ARP6* and, to a lesser degree, the *C-TAP-ARP6* transgenes are able to rescue the early flowering phenotype of *arp6-1* plants (Fig 1C) as evident by the significantly higher number of rosette leaves at the time of flowering in wild type and transgenic plants compared to *arp6-1* plants (Fig 1D). Finally, all transgenic plants showed full complementation for the loss of apical dominance and fertility defects of *arp6-1* mutant plants (Fig 1E and 1F). Overall, we conclude that the *N-TAP-ARP6* and *C-TAP-ARP6* transgenes are fully functional and thus suitable for affinity purification.

### Affinity purifications of ARP6-TAP-tag protein co-purify known components of the SWR1 complex and additional proteins

Since the *N-TAP-ARP6* and *C-TAP-ARP6* transgenes were fully functional, we proceeded with the affinity purification experiments using two independent *N-TAP-ARP6* transgenic lines (*N-TAP 11–4* and *N-TAP 1–2*) and one *C-TAP-ARP6* line (*C-TAP 10–2*). We followed the protocol described by Van Leene and colleagues to purify and elute the ARP6-TAP-tag interacting proteins and identified the eluted proteins by liquid chromatography coupled with tandem mass spectrometry (LC-MS/MS) [45]. All eluted proteins detected in our three TAP-tag experiments are listed in S1 Table. Using the database of non-specific binders of the GS^rhino^ TAP-tag, we eliminated many proteins from this list as false positives and compiled a list of ARP6-interacting proteins. Among these proteins, we identified ARP6, SWC2, SWC4, SWC6, PIE1, RuvB1, RuvB2, ACTIN1, ARP4, YAF9a, and H2A.Z proteins as Arabidopsis homologs of all 11 conserved subunits found in both yeast SWR1 and mammalian SRCAP complexes (Table 1). While we were able to detect HTA9 or HTA11 in all of our TAP-tag experiments, we never detected HTA8, the third member of the H2A.Z family. This finding is perhaps not surprising considering the fact that HTA8 has the lowest expression in all tissues when compared to the other H2A.Z family members in Arabidopsis (S1A Fig). We also did not detect the YAF9b protein (Table 1) even though Arabidopsis YAF9a and YAF9b have been previously shown to act redundantly and are required for proper *FLC* expression [44, 46, 47]. In addition to H2A.Z, we identified three H2B histones in our TAP experiments: HTB2, HTB4, and HTB9 (Table 1). Since H2A.Z histones are deposited into nucleosomes as H2A.Z/H2B dimers, we sought to investigate whether specific H2A.Z proteins might have preferential H2B partners. If this is true, we would expect to see synchronized expression of specific H2A.Z/H2B pairs in various Arabidopsis tissues. Using publicly available microarray expression data [48] for the two H2A.Z and three H2B histones that we identified, we observed that HTA11 and HTB2 had highly similar expression profiles across tissues (S1B Fig), while the expression of HTA9 matched very well with HTB4 expression and, to a slightly lesser degree, with HTB9 expression (S1C and S1D Fig). These results indicate that the Arabidopsis H2A.Z histones may have preferential H2B partners when deposited as dimers into nucleosomes.

Interestingly, in addition to known subunits of the SWR1 complex, we also reproducibly identified several other nuclear proteins as being associated with the SWR1 complex (Table 1). These include MBD9, a protein with a methyl-CpG-binding domain and various chromatin-binding domains [49–51], TRA1A and TRA1B, two subunits in the SPT module of an Arabidopsis SAGA complex [52] that are also homologs of the yeast NuA4 subunit Tra1 and the mammalian TIP60 subunit TRRAP [34, 44], and three members of a plant-specific Alfin1-like family (AL5, AL6, and AL7) best known for their regulation of abiotic stress responses in Arabidopsis and ability to bind di- and tri-methylated lysine 4 of histone H3 (H3K4me2/3) [53, 54].

**Table 1:** *Arabidopsis thaliana* homologs of SWR1-C subunits co-purify with N-TAP ARP6 and C-TAP ARP6.

IP-ed proteins	Gene ID	Predicted MW (kDA)	Peptide number (N-TAP 11–4)	Peptide number (N-TAP 1–2)	Peptide number (C-TAP 10–2)
**ARP6** **PIE1** **SWC2** **SWC4** **SWC6** **RuvB1** **RuvB2** **HTA8** **HTA9** **HTA11** **HTB2** **HTB4** **HTB9** **ARP4** **ACT1** **YAF9a** **YAF9b** **MBD9** **TRA1A** **TRA1B** **AL5** **AL6** **AL7**	**AT3G33520** **AT3G12810** **AT2G36740** **AT2G47210** **AT5G37055** **AT5G22330** **AT5G67630** **AT2G38810** **AT1G52740** **AT3G54560** **AT5G22880** **AT5G59910** **AT3G45980** **AT1G18450** **AT2G37620** **AT5G45600** **AT2G18000** **AT3G01460** **AT2G17930** **AT4G36080** **AT5G20510** **AT2G02470** **AT1G14510**	**46.9** **234** **42.1** **49.8** **19.1** **50.3** **52.1** **14.4** **14.3** **14.5** **15.7** **16.4** **16.4** **48.9** **41.8** **30.2** **30.4** **240.4** **436.3** **433.6** **29.6** **28.8** **28.3**	**34** **28** **5** **7** **2** **25** **23** **ND** **2** **ND** **6** **8** **8** **14** **9** **1** **ND** **12** **18** **17** **3** **4** **3**	**35** **12** **3** **5** **3** **22** **22** **ND** **ND** **1** **ND** **5** **ND** **11** **9** **ND** **ND** **4** **1** **1** **2** **2** **ND**	**28** **31** **3** **5** **3** **21** **23** **ND** **ND** **3** **5** **5** **5** **13** **8** **6** **ND** **6** **15** **11** **2** **2** **3**

We used mass spectrometry (MS) analysis to identify eluted proteins co-purified with ARP6-TAP-tag. All proteins identified by MS from three independent experiments (S1 Table) were first filtered against the proteins purified from WT by TAP and then against the list of 760 known non-specific binders of the GS-TAP-tag. Table 1 contains the proteins that are not found on the list of the TAP-tag background proteins and are known homologs of SWR1 complex subunits in yeast and SRCAP subunits in mammals. The table also includes proteins such as MBD9, TRA1A and TRA1B, and Alfin1-like family proteins (bottom six rows) that may represent novel subunits of the SWR1 complex. The table shows the expected MWs for each identified protein, and the total number of peptides identified by MS from two *N-TAP-ARP6* and one *C-TAP-ARP6* transgenic plants. ND = not detected.

### MBD9 is required for H2A.Z incorporation into chromatin

MBD9, a methyl-CpG-binding domain-containing protein, was identified in all three TAP-tag experiments as an ARP6-interacting partner (Table 1). Previous studies have shown that *mbd9* mutants flowered significantly earlier than WT plants, due to reduced *FLC* expression, and produced more inflorescence branches when compared to WT plants [50], which are phenotypes also found in *arp6* mutants [40]. We discovered that, in addition to the above-mentioned defects, *mbd9* plants have serrated rosette leaves and a significantly increased number of flowers with extra petals (S2 Fig), which are phenotypes also associated with the loss of *ARP6* [18, 39, 40]. Furthermore, examination of the previously reported MBD9 enrichment pattern at the *FLC* locus revealed that MBD9 occupied the two *FLC* regions previously shown to also have the highest H2A.Z enrichment in WT plants [41, 51]. Given that *arp6* and *mbd9* plants have similar phenotypes, that H2A.Z and MBD9 appear to occupy the same sites at the *FLC* locus, and that MBD9 co-purified with ARP6 in our TAP-tag experiments, we investigated whether MBD9 plays a role in the incorporation of H2A.Z into chromatin. We performed three biological replicates of chromatin immunoprecipitation coupled with high-throughput sequencing (ChIP-seq) using an H2A.Z antibody [41] on WT, *arp6-1*, and *mbd9-1* seedlings. At least 8 million high-quality, nuclear reads were aligned to the TAIR10 genome for each replicate, resulting in thousands of reproducible H2A.Z peaks for each genotype (S3A Fig). Spearman correlation analysis indicated a high degree of overlap among H2A.Z ChIP replicates for a given genotype with the exception of *mbd9-1* replicate 3, which correlated moderately with other two *mbd9* replicates (S3B Fig).

**Fig 2:**
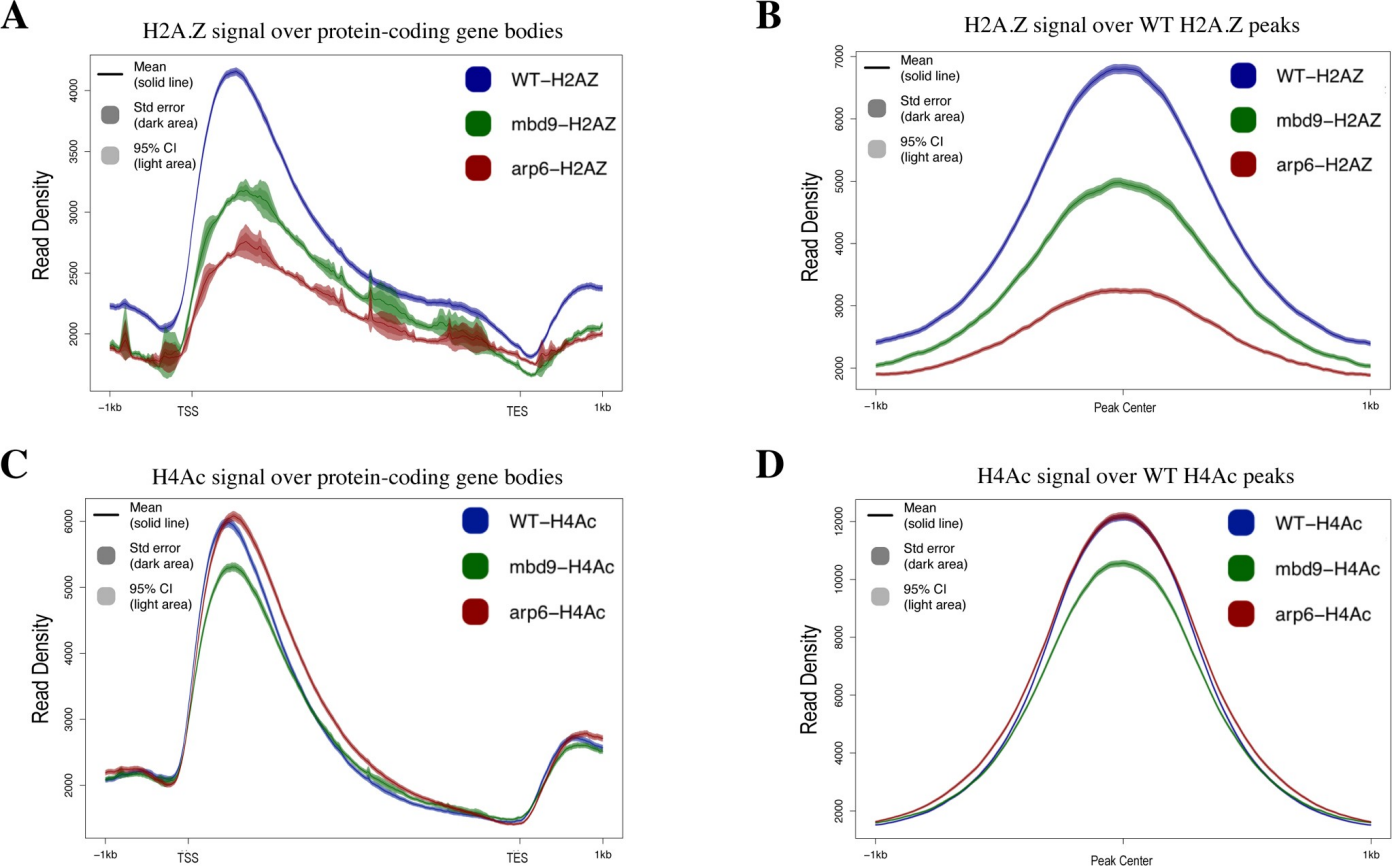
The effects of MBD9 on H2A.Z incorporation and H4 acetylation. All ChIP-seq profiles of WT (shown in blue), *mbd9-1* (shown in green), and *arp6-1* (shown in red) were produced using SeqPlots. Standard error is represented as shading around the solid line of the mean signal. (A) Average ChIP-seq H2A.Z profiles plotted over gene body coordinates for all protein-coding Arabidopsis genes, from the transcript start site (TSS) to the transcript end site (TES). (B) H2A.Z signal for each genotype over reproducible H2A.Z-enriched regions from WT plants. (C) Average ChIP-seq H4Ac profiles plotted across gene bodies. (D) H4Ac signals over reproducible H4Ac-enriched regions from WT plants.

The average H2A.Z enrichment profile (the average of all three scaled replicates) across all gene bodies in WT plants showed the highest enrichment of H2A.Z just after the transcription start site, with decreasing enrichment toward the 3’ end, as expected (Fig 2A). The pattern of H2A.Z enrichment across genes in *arp6-1* showed a similar profile but with extremely reduced enrichment, while *mbd9-1* plants had an intermediate level of H2A.Z enrichment between WT and *arp6-1* (Fig 2A). To analyze all of the regions normally enriched for H2A.Z, we identified peaks of H2A.Z enrichment that were present in at least two of the three H2A.Z ChIP-seq replicates in WT plants, and we then examined H2A.Z levels at these same sites in *arp6-1* and *mbd9-1* mutants. As observed for gene bodies, the 7039 sites reproducibly enriched for H2A.Z in WT were nearly depleted of H2A.Z in *arp6-1*, while there was an intermediate H2A.Z depletion in *mbd9-1* plants (Fig 2B). Western blotting for H2A.Z on acid-extracted nuclear proteins isolated from WT, *arp6-1*, and *mbd9-1* showed a decrease in H2A.Z levels in both *arp6-1* and *mbd9-1*, suggesting that the lack of chromatin incorporation may lead to H2A.Z degradation (S4A and S4B Fig). To test whether the observed reduction of H2A.Z incorporation might be due to either SWR1 components or *H2A*.*Z* genes being misregulated in *mbd9* plants, we performed qRT-PCR experiments to measure the expression of *PIE1*, *ARP6*, *MBD9*, *SWC4*, *SWC6*, and *YAF9a*, as well as *HTA8*, *HTA9*, and *HTA11* genes, in WT, *arp6-1*, and *mbd9-1* plants. We found that the expression of these genes was not substantially altered and, therefore, unlikely to account for the observed depletion of H2A.Z levels in *mbd9-1* plants (S5 Fig). Using RNA-seq, a related study has also demonstrated that the expression of SWR1 subunit genes was largely unaffected in *mbd9-3* plants (Potok et al. 2019, bioRxiv 10.1101/657296). Overall, these results indicate that MBD9 is indeed required for proper H2A.Z incorporation into chromatin.

To further confirm our results with respect to the role of MBD9 in H2A.Z deposition, we performed ChIP-qPCR experiments using WT, *arp6-1*, *mbd9-1*, and two additional *mbd9* T-DNA alleles, *mbd9-*2 *and mbd9-*3 [50]. We first assayed H2A.Z abundance at two distinct regions of the *FLC* gene: the first and last exon (regions 2 and 9, respectively, as described in [41]). Regions 2 and 9 are the sites on the *FLC* gene where H2A.Z is most highly enriched in WT plants, and that enrichment is lost in *arp6-1* mutant plants (S6A Fig, [41]). We found that in plants homozygous for any of the three *mbd9* alleles, the amount of H2A.Z at *FLC* regions 2 and 9 was reduced at least 2-fold when compared to WT plants (S6A Fig), indicating that MBD9 contributes to H2A.Z deposition at the *FLC* gene. We also measured the H2A.Z abundance at *ASK11* and *At4*, two phosphate starvation response genes previously shown to have H2A.Z deposited in their chromatin [55]. We discovered that in *mbd9* plants these genes were depleted of H2A.Z to similar levels as in *arp6-1* plants when compared to WT (S6B Fig). Taken together, our results indicate that MBD9 is required for proper H2A.Z levels at multiple Arabidopsis genomic loci and is, therefore, functionally related to the SWR1 complex.

As MBD9 was previously reported to have histone acetyltransferase (HAT) activity in vitro and was found to associate with acetylated H4 [51], we also examined the global levels of histone H4 N-terminal acetylation (H4Ac) in WT, *mbd9-1*, and *arp6-1* plants. At least 5 million high-quality, nuclear reads were aligned to the TAIR10 genome for each replicate, resulting in thousands of reproducible, H4Ac peaks for each genotype (S3A Fig). Spearman correlation analysis indicated a high degree of overlap among H4Ac ChIP replicates for a given genotype with the exception of *mbd9-1* replicate 3, which correlated moderately with other two *mbd9-1* replicates due to having the lowest levels of H4Ac among the replicates (S3C and S7 Figs). If MBD9 is responsible for global acetylation of H4, as previously reported [51], we would expect to see a dramatic reduction of acetylated H4 in *mbd9-1* mutants compared to WT. However, we found that the average genome-wide distribution of acetylated H4 was only modestly reduced in *mbd9-1* plants, while *arp6-1* plants had H4Ac levels similar to WT when examined across all gene bodies or at all sites enriched for acetylated H4 in WT plants (Fig 2C and 2D). Western blot analysis using H4Ac antibodies on acid-extracted nuclear proteins from WT, *arp6-1*, and *mbd9-1* plants was consistent with the ChIP-seq findings that *mbd9-1* plants have moderately reduced levels of H4Ac when compared to WT (S4A and S4C Fig). Collectively, our ChIP-seq results demonstrate a major role for MBD9 in maintaining proper H2A.Z levels. MBD9 may also have a minor role in the acetylation of H4, but this is likely to be indirect given that the protein does not contain an identifiable acetyl CoA-binding or acetyltransferase domain.

### A subset of H2A.Z-enriched sites require MBD9 for H2A.Z incorporation

**Fig 3:**
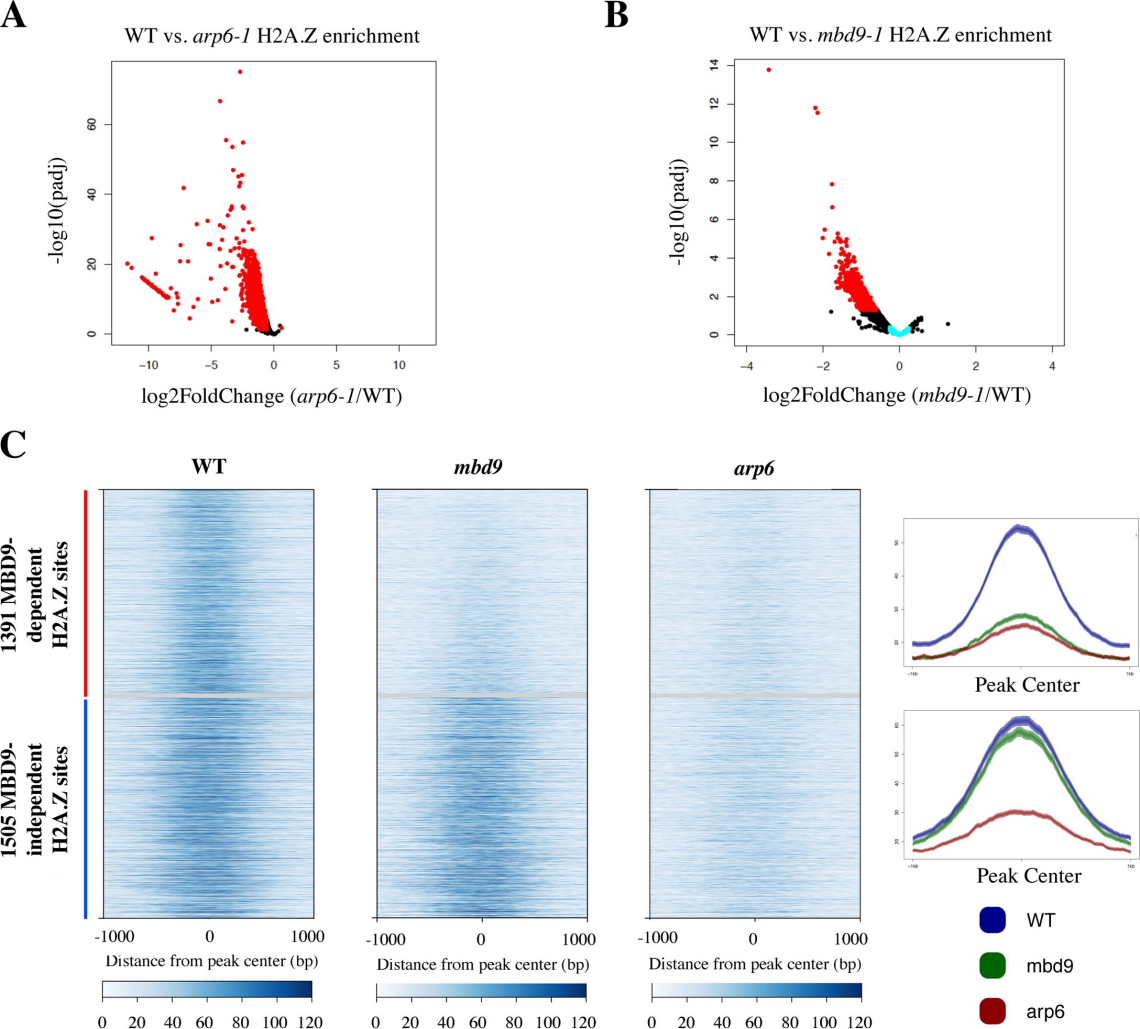
Identification of 1391 H2A.Z-enriched sites that require MBD9 for H2A.Z incorporation into chromatin. (A-B) Volcano plots of–log10 of the adjusted P value (y-axis) versus log2 fold change of H2A.Z ChIP-seq reads (x-axis) between wild type plants and *arp6-1* mutant plants (A) or *mbd9-1* mutant plants (B). Each point corresponds to a called H2A.Z peak that was analyzed by DESeq2. Peaks that had a log2 fold change equal to or greater than 0.6 and an adjusted p value of 0.05 or less are colored red. Out of 7,039 peaks analyzed, there are 6,266 peaks that are significantly depleted of H2A.Z in *arp6-1* plants (A, red dots), and 1,391 peaks that are significantly depleted of H2A.Z in *mbd9-1* plants (MBD9-dependent peaks, panel B, red dots). Peaks that had an absolute log2 fold change from 0 to 0.25 in the WT to *mbd9-1* comparison are colored light blue (MBD9-independent peaks, panel B, light blue dots). (C) Heatmaps (left) and average plots (right) of the 1391 MBD9-dependent (red bar, top of heatmaps, top average plot) peaks that are significantly depleted of H2A.Z in *mbd9-1* plants and 1,505 MBD9-independent (blue bar, bottom of heatmaps, bottom average plot) peaks that had an absolute log2 fold change from 0 to 0.25 in the WT to *mbd9-1* plants. Plots are centered on each peak and show a 2 kb window around the peak centers.

The intermediate loss of H2A.Z in *mbd9-1* plants compared with *arp6-1* plants (Fig 2A and 2B) suggested that MBD9 may be required for incorporation of H2A.Z at only a subset of H2A.Z-enriched sites or it may be required for complete H2A.Z deposition at all sites. To identify genomic regions that require MBD9 for H2A.Z incorporation into chromatin, we quantified the normalized H2A.Z ChIP-seq read abundance from WT, *arp6-1*, and *mbd9-1* mutant plants across all of the H2A.Z-enriched regions that were reproducibly identified in the WT replicates. We performed DESeq analysis [56] to quantitatively compare WT to *mbd9-1* and WT to *arp6-1* H2A.Z levels at each site (S2 Table). H2A.Z levels were significantly depleted in *arp6-1* at nearly all of the H2A.Z sites, as expected for a mutation that disrupts the SWR1 complex (Fig 3A). In contrast, out of the 7039 H2A.Z-enriched sites, we identified only 1391 sites that had reduced H2A.Z by at least 1.5-fold (log2 fold change of at least 0.6 with an adjusted p value ≤ 0.05) in *mbd9-1* compared to WT (Fig 3B).

To further examine the nature of the H2A.Z deposition defect in *mbd9* mutants, we visualized H2A.Z enrichment and distribution across the 1391 sites that lose H2A.Z in *mbd9-1*, which we refer to as MBD9-dependent H2A.Z sites. For comparison, we selected a similarly sized set of MBD9-independent H2A.Z sites (1505 sites with an average fold difference of less than 1.19 between WT and *mbd9-1*, which is an absolute log2 fold change of less than 0.25). This analysis revealed a drastic reduction in H2A.Z occupancy at each of the MBD9-dependent H2A.Z sites when comparing WT and *mbd9-1*, but with maintenance of the same overall pattern of occupancy (Fig 3C). The level of H2A.Z in *mbd9-1* at MBD9-dependent sites was depleted to levels similar to *arp6-1*. In contrast, the MBD9-independent H2A.Z sites showed equivalent profiles and occupancy levels between WT and *mbd9-1*, but still showed reduced H2A.Z in *arp6-1* (Fig 3C). To further validate these results, we chose three MBD9-dependent and three MBD9-independent H2A.Z sites and performed qPCR experiments using the same H2A.Z ChIP material from WT and *mbd9-1* plants previously utilized for ChIP-qPCR analysis of *FLC*, *ASK11*, and *At4* genes. We found that all three MBD9-dependent loci showed statistically significant depletion of H2A.Z in *mbd9-1* when compared to WT (S8A and S8B Fig), while none of the three MBD9-independent sites had significantly different levels of H2A.Z between *mbd9-1* and WT plants (S8C and S8D Fig). Thus, we conclude that MBD9 is required for proper H2A.Z deposition at a subset of the sites that this histone variant normally occupies.

### MBD9-dependent H2A.Z sites have chromatin features distinct from MBD9-independent H2A.Z sites

In order to understand why MBD9 is required for H2A.Z deposition at certain sites and not others, we first analyzed the genomic distribution of MBD9-dependent H2A.Z sites compared to the MBD9-independent H2A.Z sites. We found that the two sets are distributed similarly across the genome, with more than 80% of each set of coordinates localizing within genic regions (S9A Fig). Both MBD9-dependent and MBD9-independent H2A.Z sites were found primarily at the 5’ end of protein-coding gene (PCG) bodies, with the MBD9-dependent sites found slightly more upstream of the MBD9-independent sites (S9B and S9C Fig). PCGs nearest to the MBD9-dependent sites had reduced H2A.Z levels throughout the entire gene body in *mbd9-1* and *arp6-1* plants, whereas PCGs nearest to the MBD9-independent sites had similar gene body H2A.Z levels in WT and *mbd9-1*, but still had reduced H2A.Z levels in *arp6-1* (S9C Fig). For the 1322 PCGs found nearest to the MBD9-dependent H2A.Z sites (S2 Table) there was no significantly overrepresented Gene Ontology (GO) terms identified using either of the two different GO analysis tools (see methods), indicating that MBD9-mediated deposition of H2A.Z is not associated with functionally-related gene sets or a particular cellular pathway.

**Fig 4:**
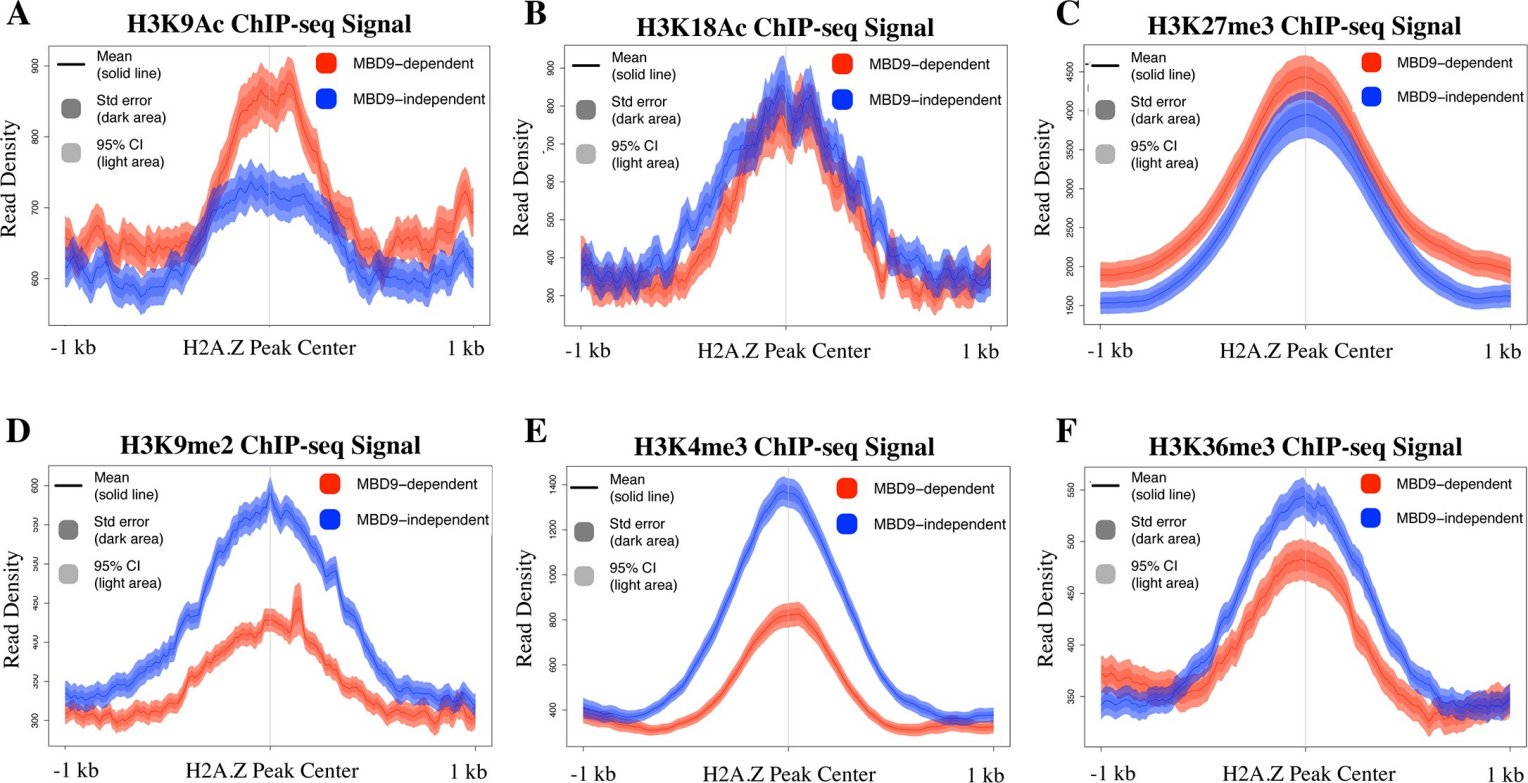
H2A.Z sites that require MBD9 have distinct chromatin properties. Average ChIP-seq profiles of H3K9Ac (A), H3K18Ac (B), H3K27me3 (C), H3K9me2 (D), H3K4me3 (E), and H3K36me3 (F) at MBD9-dependent H2A.Z sites (shown in red) and MBD9-independent H2A.Z sites (shown in blue). Plots show a 2 kb window around the center of the MBD9-dependent and MBD9-independent sites. Average read density for either set of sites is shown as a single line depicting the mean with standard error denoted as dark shading, and 95% confidence interval denoted as light shading.

We also examined various histone modification profiles at the two types of sites using publicly available ChIP-seq data from WT plants in order to discern any differences between H2A.Z sites that require MBD9 and those that do not. Interestingly, we found that in WT plants the average level of histone H3 lysine 9 acetylation (H3K9Ac) is higher at MBD9-dependent H2A.Z sites than it is at the sites that do not require MBD9 (Fig 4A). However, no significant differences were found in the average enrichment of histone H3 lysine 18 acetylation or H3 lysine 27 trimethylation (H3K18Ac and H3K27me3, respectively) between the two types of loci (Fig 4B and 4C). On the other hand, we did observe less abundant enrichment of dimethylated lysine 9 of histone H3 (H3K9me2) at MBD9-dependent H2A.Z sites (Fig 4D). The same pattern was observed for H3 trimethylation at lysine 4 or lysine 36 (H3K4me3 and H3K36me3, respectively), with consistently lower levels of each mark at MBD9-dependent H2A.Z sites (Fig 4E and 4F).

To better understand the differences in chromatin profiles between the two sets of loci, ten different histone marks (H2A.Z, H4Ac, H3K27me3, H2AK121Ub, H3K9me2, H3K9Ac, H3K18Ac, H3K27Ac, H3K4me3, and H3K36me3) were plotted around a 2 kb window centered on the H2A.Z peak of either the MBD9-dependent or MBD9-independent sites (S10A Fig). The heatmaps were clustered into 4 k-means clusters and three similar sets of chromatin states were observed in both MBD9-dependent and MBD9-independent loci. These include euchromatic regions (S10B Fig), H3K27me3-rich sites likely regulated by PRC2 (S10C Fig), and sites that have H2A.Z but are relatively depleted of the other examined histone modifications. The observed average differences in specific histone marks between the two sets of loci (Fig 4) appear to be driven by increased levels of H3K9Ac at MBD9-dependent euchromatic regions (S10B Fig), decreased levels of H3K9me2 and H3K4me3 in MBD9-dependent PRC2-regulated regions (S10C Fig), as well as reductions in H3K9me2 and H3K36me3 at the MBD9-dependent regions relatively depleted of most histone modifications (S10D Fig).

Given the predominant gene-body localization of H2A.Z, the differences in histone modification levels between MBD9-dependent and -independent sites could simply reflect differences in expression levels of the underlying genes. However, using publically available RNA-seq data, we found that genes nearest to the sites in each category span a wide range of expression levels and are not significantly different from one another in terms of steady-state transcript levels (unpaired t-test, p < 0.05, S11 Fig). Thus, MBD9 may specifically recognize or be repelled by specific chromatin features, which could help guide the SWR1 complex to specific DNA sites.

### MBD9 has distinct binding preferences for modified histone tails

**Fig 5:**
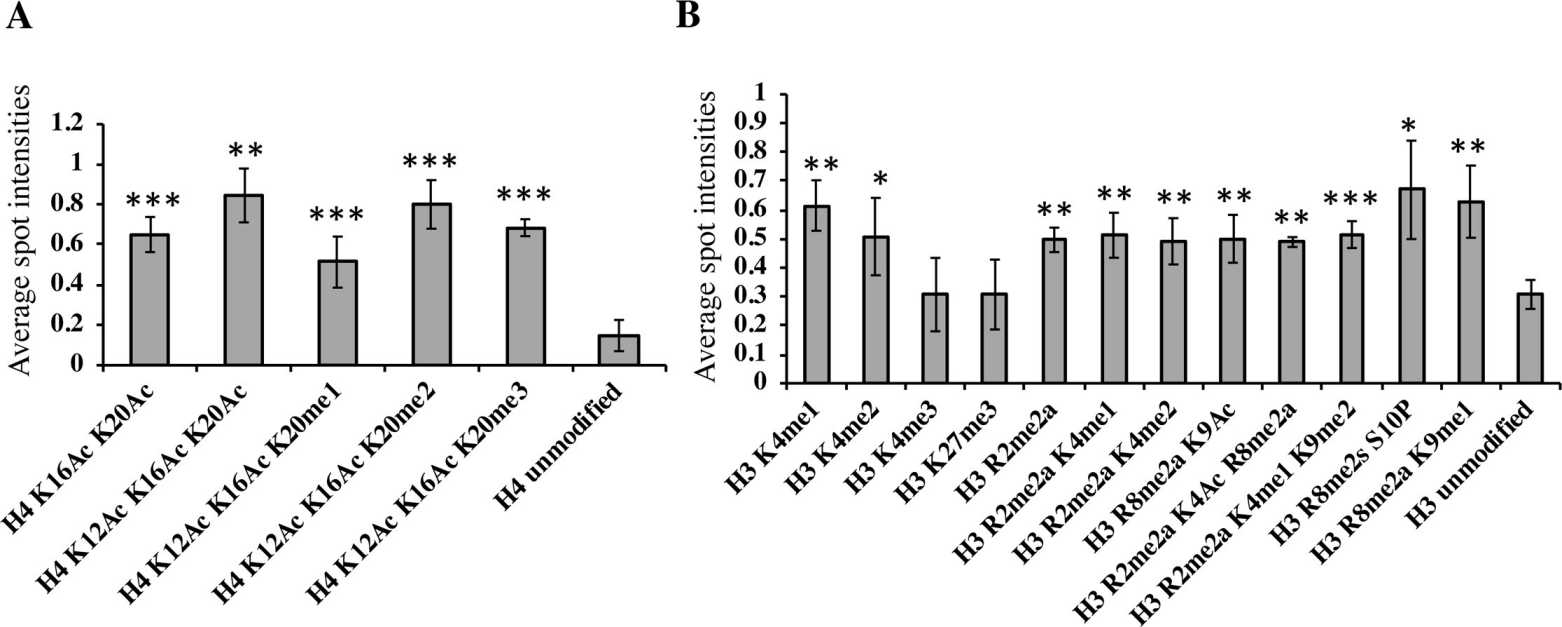
MBD9 has distinct binding preferences for modified histone tails. The intensities of each spot on the histone peptide array after interaction with a full-length MBD9 protein were quantified using software provided by the array manufacturer and were further compared to the spot intensities of unmodified H4 peptide (A), or unmodified H3 peptide (B). Y-axis represents average spot intensities with values ranging from 0 (no signal) to 1 (maximum signal strength). Each bar with standard deviations represents an average of 4 array spot intensities (two experimental replicates, each with two technical replicates) for a given modified histone peptide. Only the normalized average spot intensities with values higher than 0.49 are presented here except for the spot intensities of control peptides (unmodified H4 in panel A, and unmodified H3 peptide in panel B), and of two additional peptides in panel B (H3K4me3 and H3K27me3), which are included for comparison. Asterisks indicate level of significant differences in spot intensities between given histone peptides and control peptides (* = p<0.05, ** = p<0.01, *** = p<0.001), calculated using unpaired, one-tail t-tests. (A) MBD9 shows significant interaction preference for acetylated H4 residues. (B) MBD9 also shows higher preference for mono- and di-methylated H3K4 over tri-methylated H3K4, strongly interacts with methylated arginines in combination with other modifications of histone H3, including K9Ac, and it does not recognize H3K27me3 above background.

To examine which histone marks MBD9 may directly recognize, we performed a histone peptide array assay using the full-length MBD9 protein (S12 Fig). We found that MBD9 most strongly interacted with histone H4 peptides that were acetylated at lysine (K) residues 12, 16, and 20, or acetylated at K12 and K16 while being di- or trimethylated at K20 (Fig 5). The protein also bound to H4 peptides containing both K16Ac and K20Ac, or K12Ac and K16Ac along with K20me1. This affinity of MBD9 for acetylated H4 is consistent with a previous report [51].

Interestingly, MBD9 interacted with H3 peptides that were mono- or dimethylated at K4, but did not appear to bind the H3 peptide that was trimethylated at K4. This lack of affinity for H3K4me3 could explain the relative depletion of this mark at MBD9-dependent H2A.Z sites (Fig 4E). Other H3 peptides that showed significant binding of MBD9 include those dimethylated at arginines 2 and/or 8 (Fig 5). Collectively, these results suggest that MBD9 recognizes distinct combinations of histone modifications that are likely to influence its chromatin binding characteristics.

### MBD9 is not a core subunit of the Arabidopsis SWR1 complex

**Fig 6:**
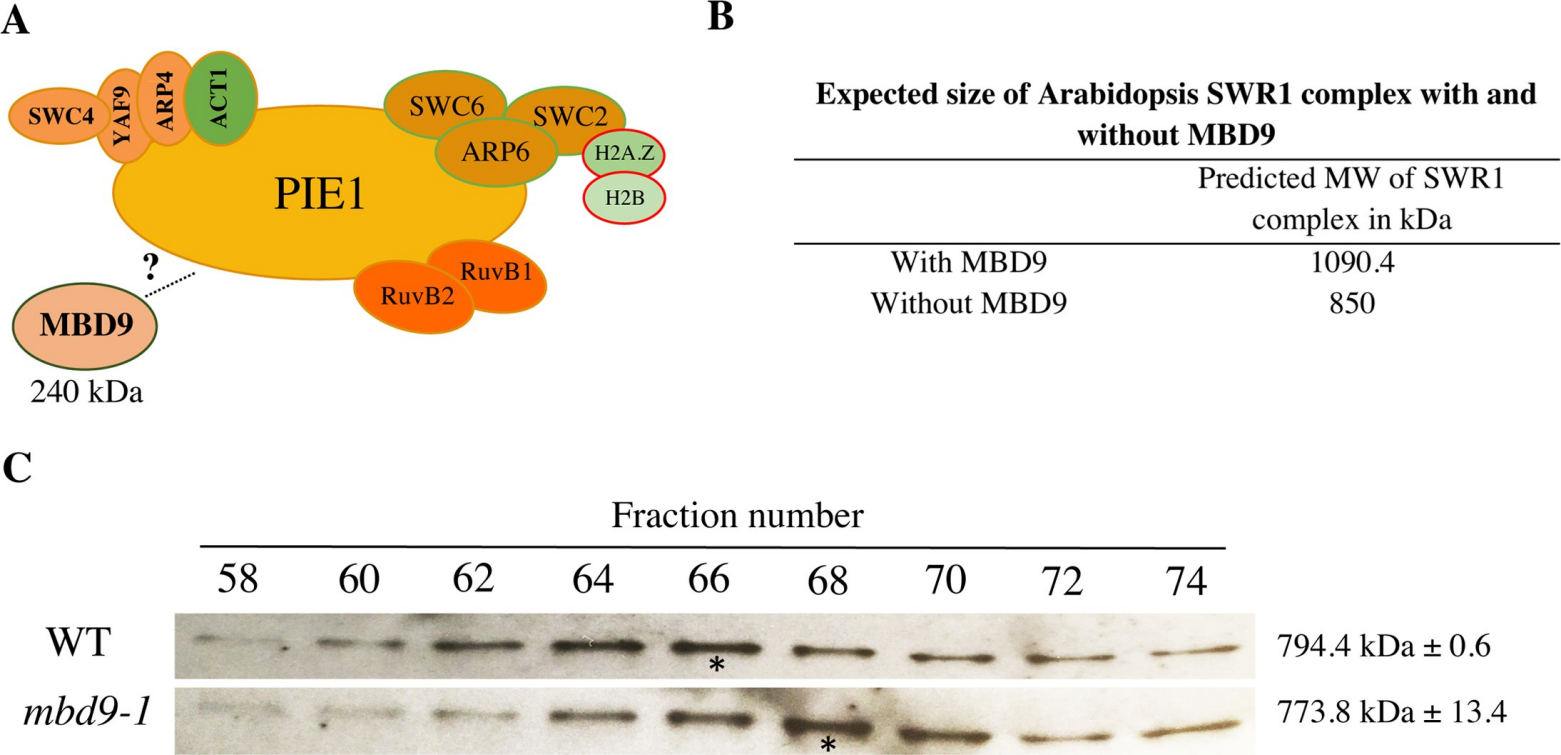
MBD9 is not a core subunit of the Arabidopsis SWR1 complex. (A) Schematic representation of the Arabidopsis SWR1 protein complex based on TAP-tag experiments, including MBD9 as a potential SWR1 subunit. (B) Estimated size of the Arabidopsis SWR1 complex with and without MBD9 as a subunit. The estimated size was calculated based on the known stoichiometry of the yeast SWR1 complex [59] and predicted molecular weights of the Arabidopsis SWR1 subunits listed in Table 1. (C) Protein gel blots of even-numbered SEC fractions from WT (top blot) and *mbd9-1* (bottom blot) plants. The blots were incubated with the ARP6 monoclonal antibody [40]. Asterisks indicate the ARP6 peak fractions. The average molecular weights of ARP6-containing protein complexes in WT and *mbd9-1* plants were calculated from two biological replicates and presented on the right side of the corresponding protein blots.

To determine whether the MBD9 protein, with an estimated molecular mass of 240 kDa, is an integral component of the Arabidopsis SWR1 complex (Fig 6A) we performed size-exclusion chromatography (SEC) experiments on protein extracts from WT and *mbd9-1* plants, followed by western blotting for ARP6. This allowed us to define the native size of the complex and to determine whether this size changes in the absence of MBD9, as would be expected if this protein were a stoichiometric component of the SWR1 complex (Fig 6B), as previously demonstrated for the PIE1 subunit [41]. Using an ARP6 monoclonal antibody [40], we detected ARP6 protein in its native form as a part of a multi-subunit complex with a molecular mass of ~800 kDa (Fig 6C). When the SEC experiments were performed on *mbd9-1* extracts, the ARP6 peak did not significantly shift and the estimated molecular mass of the native ARP6 complex in *mbd9-1* plants was ~775 kDa (Fig 6C) in two biological replicates. These results strongly suggest that MBD9 is not a core component of the ARP6-containing SWR1 complex, but most likely interacts with it in a more transient manner. Alternatively, MBD9 may be a stable component of a minor subset of SWR1 complexes.

### *arp6-1;mbd9-1* double mutant plants have a more severe phenotype than either single mutant

**Fig 7:**
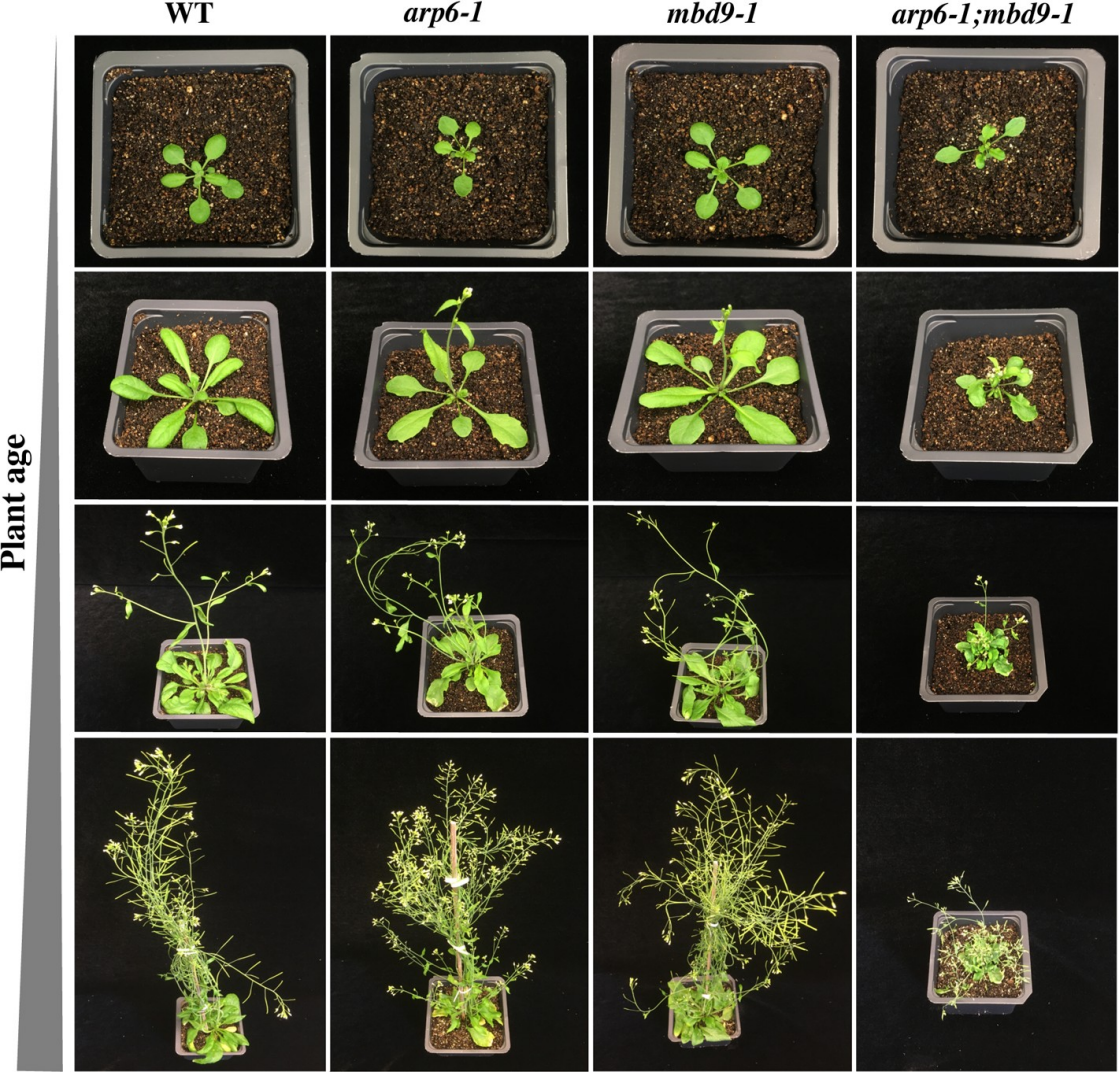
*arp6-1;mbd9-1* double mutant plants have a more severe phenotype than either single mutant. WT, *arp6-1*, *mbd9-1*, and *arp6-1;mbd9-1* plants grown under long-day conditions were individually photographed at 7, 14, 21, and 35 days after stratification. *arp6-1;mbd9-1* double mutant plants have severely delayed development and are dwarfed compared to single *arp6-1* and *mbd9-1* mutant plants.

Collectively, we discovered that MBD9 is required for proper H2A.Z deposition at many sites, but is not stably associated with the SWR1 complex. To investigate genetic interactions between *MBD9* and *ARP6*, we generated *arp6-1;mbd9-1* double mutant plants. We have shown that single *arp6-1* and *mbd9-1* mutant plants have similar phenotypic defects (S2 Fig) and that both ARP6 and MBD9 regulate H2A.Z incorporation into chromatin (Fig 2A and 2B). If these two proteins are subunits of the same complex or function exclusively in the same genetic pathway then double mutant plants should be phenotypically indistinguishable from single mutants, as previously shown for *arp6;swc6* plants [18, 37]. Instead, we observed that the double mutants displayed much more severe defects (dwarf stature, deformed leaves, and drastically reduced fertility) than the individual single mutants throughout development (Fig 7). Importantly, these phenotypes in the double mutant plants reverted back to those of each single mutant by introducing either the genomic *ARP6* or *MBD9* constructs into the double mutants (S13 Fig), indicating that these defects were truly the result of simultaneous loss of ARP6 and MBD9 functions. To investigate whether the more severe phenotype in *arp6-1;mbd9-1* plants is caused by further reduction of H2A.Z incorporation into chromatin, we performed a ChIP-qPCR experiment using H2A.Z antibody on WT, *arp6-1*, *mbd9-1*, and *arp6-1;mbd9-1* plants at *FLC* regions 2 and 9. We found that double mutant plants had similar levels of H2A.Z depletion at *FLC* when compared to *arp6-1* (S14 Fig). Collectively, our findings support the idea that MBD9 is not a core subunit of the SWR1 complex and suggest that this protein has additional functions outside of H2A.Z incorporation [57].

## Discussion

### The SWR1 complex is conserved in plants

Previous studies provided important evidence suggesting that Arabidopsis contains a SWR1-like complex that mediates incorporation of H2AZ into chromatin [6, 18, 20, 36–44]. Using the SWR1-specific subunit ARP6 as bait, we successfully purified the Arabidopsis SWR1 complex and identified all 11 conserved subunits that are also found in the yeast SWR1 and mammalian SRCAP complexes. Recently, a similar study also used ARP6 as bait to isolate the Arabidopsis SWR1 complex and identified the same proteins as our study, with the addition of AL4 (Potok et al. 2019, bioRxiv 10.1101/657296). These and our results suggest that the function and structure of the canonical SWR1 complexes that incorporate histone H2A.Z into nucleosomes have been well preserved over evolutionary timescales and may be found in all eukaryotes.

TIP60 in animals is a single multifunctional complex that combines the subunits and functions of yeast SWR1 and NuA4 complexes [34]. It appears that this merger evolved as a result of the fusion of the two major scaffolding proteins, the Swr1 ATPase of the yeast SWR1 complex and the Eaf1 protein of the yeast NuA4 complex, into a single p400-like protein. This is based on the fact that p400 contains HSA, ATPase, and SANT domains, which are found separately in the yeast Swr1 and Eaf1 proteins [34, 44]. Intriguingly, Arabidopsis PIE1 (yeast Swr1 homolog) also contains HSA, ATPase, and SANT domains, implying that PIE1 may also be an ortholog of p400. In fact, Bieuszewski and colleagues originally hypothesized that PIE1 is a scaffolding component of an Arabidopsis TIP60-like complex [34, 44]. Interestingly, TRA1A and TRA1B, two proteins that co-purified with ARP6 in our TAP experiments, were recently characterized as subunits of the SPT module of an Arabidopsis SAGA complex [52] and are homologs of the yeast NuA4 subunit Tra1 and the mammalian TIP60 subunit TRRAP, further suggesting an intimate functional relationship among Arabidopsis SWR1 and NuA4/HAT complexes (Table 1). Taken together, it is plausible that plants possess both the canonical SWR1 complex and an independent NuA4-like complex, as in yeast, and may also contain a TIP60-like complex, which is found only in animals. Future purification experiments using Arabidopsis PIE1 as bait are crucial to address the question of whether plants have two distinct PIE1-containing complexes (SWR1 and a TIP60-like), and which subunits are shared between the two complexes. The existence of two different PIE1 complexes could also explain why the phenotype of *pie1* mutant plants is distinct from that of *h2a*.*z* mutants or mutations in other SWR1 components [18, 19, 37–43].

### MBD9 may recruit the SWR1 complex to mediate H2A.Z deposition to chromatin

Based on multiple studies in many model organisms, we now have a good understanding of how the SWR1 complex incorporates H2A.Z into nucleosomes [4, 58, 59]. However, several aspects of SWR1 biology are still poorly understood, including precisely how the complex is recruited to specific chromatin regions to deposit H2A.Z. In yeast, it has been shown that NuA4-mediated acetylation of specific nucleosomal sites is important for SWR1 targeting to chromatin and H2A.Z incorporation [31–35, 60]. In addition, it has been proposed that Bdf1, a bromodomain-containing subunit of the yeast SWR1 complex, recruits the complex to chromatin by recognizing acetylated H4 tails [31, 61]. Supporting this notion, the loss of Bdf1 results in global reduction of H2A.Z in chromatin [62].

In plants, little is known about the mechanisms that target the SWR1 complex to specific chromatin loci. Recent results from the Jarillo group suggest that binding of the SWC4 subunit to AT-rich DNA elements in promoters of certain genes can recruit the Arabidopsis SWR1 complex to these chromatin regions to deposit H2A.Z [36]. However, only a subset of H2A.Z-enriched genes contain AT-rich elements in their promoters, which strongly suggests that additional mechanisms of SWR1 recruitment exist in plants. The same group has recently demonstrated that the YAF9a subunit, by interacting with acetylated histones, can recruit the SWR1 complex to a subset of SWC4 target genes [47].

What role may MBD9 play in SWR1 recruitment to specific chromatin loci? In addition to a methyl-CpG-binding (MBD) domain, which is in fact thought to bind unmethylated DNA ([63], Potok et al. 2019, bioRxiv 10.1101/657296), MBD9 contains an acetyl lysine-binding bromodomain [64], and two plant homeodomains (PHD) which may recognize methylated lysine and arginine residues [65, 66]. We showed that in *mbd9* mutant plants the level of H2A.Z incorporation is significantly reduced at a subset of H2A.Z-enriched regions (Fig 3) and that these MBD9-dependent H2A.Z loci have distinct histone modification profiles relative to MBD9-independent H2A.Z loci (Figs 4 and S10). Specifically, H2A.Z sites that are dependent on MBD9 had higher levels of H3K9Ac and lower levels of H3K4me3, H3K36me3, and H3K9me2. Using the histone peptide array assay, we demonstrated that MBD9 strongly interacts with acetylated H4, suggesting that MBD9 may recognize specific H4 acetylations in the context of other modifications. The peptide array data also revealed that MBD9 has high affinity for both symmetrically and asymmetrically di-methylated arginines in the H3 N-terminus (Fig 5). The significance of this interaction is not yet clear as there are no currently available ChIP-seq data for these modifications in Arabidopsis, and the effects of methylated arginines on chromatin architecture are only poorly understood in plants [67]. The histone modification preferences of MBD9 defined by peptide array may at least partially explain some of the differences in chromatin profiles between H2A.Z sites that require MBD9 and those that do not. For example, the H3K4me3 depletion seen in a subset of MBD9-dependent sites (S10C and S10D Fig) may be explained by the relative preference of MBD9 for binding H3K4me1 or H3K4me2, but not H3K4me3. However, the binding preferences of MBD9 appear to be complex, and there are likely one or more features that more closely define the MBD9-dependent sites (*e*.*g*. methylation of H3R2 and/or R8).

MBD9 was recently found to preferentially localize to nucleosome-depleted regions (NDRs) directly upstream of H2A.Z nucleosomes (Potok et al. 2019, bioRxiv 10.1101/657296). In yeast, SWR1 also localizes to NDRs and it is proposed that while NDR localization serves as a general recruiting mechanism for SWR1, it is the interaction between SWR1 components and the nucleosomes flanking these NDRs that define the actual sites of H2A.Z deposition [68, 69]. Collectively, the current data point to a model whereby MBD9 recognizes nucleosomes with specific modification patterns, and perhaps specific DNA sequences, and interacts with SWR1 to effect its localization to specific genes.

Although MBD9 was co-purified in all of our ARP6 TAP-tag experiments (Table 1), MBD9 appears not to be a core component of the SWR1 complex (Fig 5). Two possible conclusions about MBD9’s interaction with the SWR1 complex can be made based on these results. MBD9 may interact only transiently with components of the SWR1 complex, and is therefore detected in TAP-tag experiments as previously observed for transcription factors and cofactors that recruit Arabidopsis SWI/SNF and PRC2 complexes to specific chromatin sites [70–73]. Alternatively, MBD9 could be more tightly associated with only a subset of all SWR1 complexes in Arabidopsis. In that case, our size exclusion chromatography peak of SWR1 most likely would not show a significant mass reduction in *mbd9-1* plants when compared to WT because the loss of MBD9 would affect only a minor fraction of SWR1 complexes.

With regard to the synergistic phenotype of *arp6*;*mbd9* mutants, this could result from a further reduction of H2A.Z deposition in the double mutant compared to *arp6*, or it may be a manifestation of functions of MBD9 beyond H2A.Z deposition. These two possibilities are, of course, not mutually exclusive. While we did not observe additional H2A.Z reduction at *FLC* in *arp6*;*mbd9* compared to *arp6*, a related study found that in *arp6;mbd9* double mutant plants the level of H2A.Z incorporation into chromatin is indeed further reduced genome-wide (Potok et al. 2019, bioRxiv 10.1101/657296). However, this study also found that MBD9 strongly interacts with the ISWI family of CRCs, suggesting that MBD9 has additional nucleosome remodeling functions outside of H2A.Z deposition. While further experiments are needed to determine the precise nature of MBD9’s interaction with the SWR1 complex, it is clear that MBD9 is functionally associated with the SWR1 complex and is integral to the deposition of H2A.Z at a subset of loci.

An important point to consider is whether the specific H2A.Z loss from chromatin in *mbd9* mutants reflects a lack of targeting of SWR1 to MBD9-dependent H2A.Z sites, or whether the complex is targeted properly but H2A.Z is not retained in nucleosomes after deposition in *mbd9*. It is indeed formally possible that MBD9 plays a role in H2A.Z retention in chromatin. While an absolute resolution to this issue awaits further experimentation, the most parsimonious explanation for the present observations is that MBD9 interacts with the SWR1 enzyme complex to influence where its reaction occurs, rather than acting on the product of the reaction (an H2A.Z-containing nucleosome).

The presence of H2A.Z in chromatin has been linked to both gene activation and gene repression, but how H2A.Z affects transcription in this context-dependent manner is not clear [74, 75]. In addition, how the chromatin remodelers that deposit H2A.Z are recruited to specific chromatin loci is poorly understood. Our isolation of the Arabidopsis SWR1 complex identified unexpected proteins that co-purified with this complex, including MBD9 and three members of the plant-specific Alfin family, each of which contain known modified histone-binding domains. Based on our results and data from other studies, we propose that these SWR1-associated proteins are involved in the recruitment of the complex to chromatin to incorporate H2A.Z at specific loci. In this view, the H2A.Z landscape reflects the collective effects of inherent SWR1 targeting as well as SWR1’s association with a variety of adaptor proteins, such as MBD9. With the identification of these SWR1-associated proteins, we can now start to address important mechanistic questions about the activity of SWR1 in plants and how MBD9, and perhaps Alfin1-like family proteins, may modulate SWR1 functions.

## Materials and methods

### Plant material, growth conditions, and transformation

*Arabidopsis thaliana* of the Columbia (Col-0) ecotype was used as the wild type reference, and all mutant seeds are of the Col-0 ecotype. The *arp6-1* (SAIL_599_G03), and *mbd9-1* (SALK_054659), *mbd9-2* (SALK_121881) and *mbd9-3* (SALK_039302) alleles were described previously [40, 50]. Seedlings were grown in either soil, half-strength Murashige and Skoog (MS) liquid media [76], or on half-strength MS media agar plates, in growth chambers at 20°C under a 16 hour light/8 hour dark cycle. Plasmids containing the *N-TAP-ARP6*, *C-TAP-ARP6*, and *gMBD9* constructs, driven under endogenous *ARP6* and *MBD9* promoters, respectively, were introduced into *Agrobacterium tumefaciens* GV3101 strain by electroporation. Plants were transformed with these constructs via the floral dip method [77]. Primary transgenic plants were selected on half-strength MS media agar plates containing 50 mg/L hygromycin and 100 mg/L timentin, and then transferred to soil. Two to three grams of sterilized WT seeds and *T*_*3*_ seeds homozygous *for N-TAP-ARP6* and *C-TAP-ARP6* constructs were germinated for 6 days in flasks containing 600 ml of half-strength MS media with constant shaking on rotating platform (80–90 rpm). After 6 days, the germinated seedlings were filtered to remove the excess liquid, and 50 grams of seedling tissue was frozen in liquid nitrogen and stored at -80°C.

### Plasmid DNA constructs

To construct the ARP6-TAP-tag, we fused genomic *ARP6* sequence to the tandem affinity purification (TAP) GS^rhino^ tag, recently developed for efficient affinity purifications of protein complexes in plants [45]. Gateway–compatible plasmids containing either a C-terminal TAP-tag (pEN-R2-GS_rhino-L3, [45]) or an N-terminal TAP-tag (pEN-L1-NGS_rhino-L2, [45]) were used to produce the *C-TAP-ARP6* and the *N-TAP-ARP6* constructs, respectively. To generate the *C-TAP-ARP6* construct, a total of six primers were used in three overlapping PCR reactions to produce a ~4.7 kb *attB* PCR fragment. This PCR product contained ~4.1 kb of the genomic *ARP6* sequence (from -2040 bp upstream of the start codon to +2083 bp downstream from the start codon), ~600 bp of the TAP-tag sequence fused to the C-terminal end of the *ARP6* gene, and *attB* adapters at the 5’ and 3’ ends of the PCR product for Gateway cloning. This PCR fragment was subcloned into pDONR221 gateway plasmid via BP recombination reaction using BP clonase II enzyme (Invitrogen). The construct was verified by sequencing and further sub-cloned into the destination gateway plasmid pMDC99 [78] using the LR clonase II enzyme in LR recombination reaction (Invitrogen). Similarly, the *attB N-TAP-ARP6* construct was first produced using six PCR primers in overlapping PCR reactions containing the same genomic *ARP6* DNA fragment as in the *C-TAP-ARP6*, with the TAP-tag fused at the N-terminal end of the *ARP6* gene. This PCR fragment was then sub-cloned into pDONR221 via BP reaction, verified by sequencing, and finally sub-cloned into the pMDC99 destination plasmid via LR reaction.

To generate *gMBD9* construct, which was used to transform *arp6-1;mbd9-1* double mutant plants, we first PCR-amplified 11,311 bp of genomic *MBD9* sequence (from –1936 bp upstream of the start codon to + 9372 downstream from the start codon) using *gMBD9* sequence-specific primers with *attB* adapters at their 5’ ends. The *attB* PCR product was then sub-cloned into pDONR221 gateway plasmid via BP recombination reaction (Invitrogen), verified by sequencing, and finally sub-cloned into the destination gateway plasmid pMDC99 [78] using LR recombination reaction (Invitrogen).

To clone the N-terminal *Myc-MBD9* construct into pT7CFEChis vector (Thermo Scientific), we designed two sets of primers: first set was used to PCR-produce an N-terminal Myc-tag fused in frame with the full length *MBD9* cDNA, while second primer pair was used to PCR-amplify the full pT7CFEChis vector. The two PCR products were then used in a Gibson Assembly reaction (New England Biolabs) to clone *Myc-MBD9* at an *NdeI* restriction cloning site in the pT7CFEChis plasmid to achieve a maximum expression of a fusion protein in this system. The cloning was verified by sequencing.

### Crosses and genotyping

To produce *arp6-1;mbd9-1* double mutant plants, pollen from *arp6-1* plants was used to manually pollinate *mbd9-1* plants. Since *ARP6* and *MBD9* genes are both on chromosome 3, we were only able to identify the F_2_ plants that were homozygous for one T-DNA allele and heterozygous for the other. We used F_3_ seeds from *arp6-1/arp6-1;mbd9-1/+* plants to identify the double mutant plants.

### Protein gel blotting

The proteins for western blot detection shown in Fig 1A were extracted from ~100 mg of 6-day old whole transgenic seedlings homozygous for the *arp6-1* allele and either the *C-TAP-ARP6* or *N-TAP-ARP6* constructs by first making a crude nuclei preparation using Nuclei Purification Buffer [79]. The nuclei pellets were then resuspended in 2 volumes of 1x Laemmli’s sample buffer (125 mM Tris-HCl pH 6.8, 4% SDS, 30% glycerol, and 1% β-mercaptoethanol) prior to heating and loading on a gel. For ARP6 detection on fractions from SEC experiments (see below), the eluted proteins were isolated by adding 20 μl of the StrataClean resin (Agilent) to 1 ml of each SEC fraction, incubating for 20 minutes at room temperature (RT) on a rotating platform, and then spinning down for 2 minutes at 5,000g at RT. The pelleted proteins were resuspended in 20 μl of 1x Laemmli’s sample buffer. The total proteins for western blot detection shown in S4 Fig were isolated from ~150 mg of young leaves using acid extraction protocol (see below) and the pelleted proteins were resuspended in 50 μl of 1x Laemmli’s sample buffer. The proteins were then separated on 4–20% Novex WedgeWell tris-glycine gel (Invitrogen) and transferred to Amersham nitrocellulose blotting membrane (GE Healthcare). After blocking overnight in PBST buffer (137 mM NaCl, 2.7 mM KCl, 10 mM Na_2_HPO4, 2 mM KH_2_PO4, and 0.05% tween20) containing 5% non-fat dry milk, the blots were incubated with primary antibody (1:100 dilution for monoclonal mouse ARP6 antibody [40], 1:2,000 dilution for peroxidase anti-peroxidase (PAP) soluble complex antibody (Sigma-Aldrich) that detects the TAP-tag, 1:1000 dilution for H2A.Z antibody [41], and 1:500 dilution for H4Ac antibody (Millipore 06–866)) in blocking solution for 1 hour at RT. The blots were washed 3 times for 5 minutes in PBST. The ARP6 blot was then incubated with the anti-mouse horseradish peroxidase (HRP)-conjugated secondary antibody (1:2,000 dilution, GE Healthcare), while H2A.Z and H4Ac blots were incubated with anti-rabbit HRP-conjugated secondary antibody (1:3,000 dilution, GE healthcare). The ARP6, H2A.Z, and H4Ac blots were washed 3 more times for 5 minutes in PBST, and all blots were then incubated with ECL detection reagents for 2 minutes (Thermo Scientific). The ARP6 blots and PAP blot were exposed to Amersham Hyperfilm ECL (GE Healthcare) to detect protein bands, while H2A.Z and H4Ac blots were scanned for chemiluminescence signal using ChemiDoc MP imaging system instrument (BioLab).

### Acid extraction of proteins for western blotting

Approximately 150 mg of young leaves from WT, *mbd9-1*, and *arp6-1* plants were ground to a fine powder, homogenized in 3 ml of histone extraction buffer (0.25 M sucrose, 1 mM CaCl_2_, 15 mM NaCl, 60 mM KCl, 5 mM MgCl_2_, 15 mM PIPES pH 7.0, 0.5% Triton X-100 with protease inhibitors cocktail (Roche) and 10 mM sodium butyrate), filtered through the 70 micron strainer, and incubated for 15 minutes at 4°C on a nutator. After centrifugation at 4,500g for 20 minutes at 4°C, the pellets were resuspended in 500 μl of 0.1 M H_2_SO_4_ and incubated overnight at 4°C on a nutator. After centrifugation for 10 minutes at 17,000g, total proteins were precipitated from the supernatant with concentrated trichloroacetic acid to a final concentration of 30%. The protein pellets were washed twice with an acetone solution containing 0.1% HCl and then once with acetone. The protein pellet was then air-dried and resuspended in 50 μl of 1x Laemmli’s sample buffer.

### Expression of the myc-tagged full-length MBD9 protein

Myc-MBD9 fusion protein, cloned into the pT7CFEChis plasmid, was expressed using the 1-step human coupled *in-vitro* translation (IVT) kit (Thermo Scientific) following manufacturer’s recommendations. In parallel, a GFP protein (pT7CFEcHis plasmid carrying GFP; included in the kit) was expressed and served as a positive control for IVT reaction. The expression of GFP was confirmed by visualizing GFP on a fluorescence microscope (Olympus) using 2 μl of an IVT reaction, while the expression of Myc-MBD9 was confirmed by western blotting using 4 μl of an IVT reaction and an anti-myc antibody to detect the 245 kDa protein (S12 Fig).

### Histone peptide array assay

Two MODified peptide array slides (Active Motif) were first briefly washed in PBST buffer and then blocked in 5% milk-PBST buffer for 1 hour at RT. After blocking, the slides were washed three times for 5 minutes in PBST. One slide was then incubated with 16 μl of the IVT Myc-MBD9 protein, while the other slide was incubated with 16 μl of the IVT GFP protein, both diluted in 8 ml of the binding buffer (50 mM HEPES pH 7.5, 100 mM NaCl, 1 μM DTT, and 50% glycerol) overnight at 4°C on a nutator. Next day, the slides were washed three times for 5 minutes in PBST buffer and then incubated with a primary anti-myc antibody (1:2,000 dilution, Abcam) for 1 hour at RT. The slides were washed again three times for 5 minutes in PBST and then incubated with anti-mouse HRP-conjugated secondary antibody (1:5,000 dilution, GE Healthcare) for 30 minutes at RT. The slides were incubated with ECL detection reagents for 2 minutes (Thermo Scientific) and scanned with the ChemiDoc MP imaging system (BioRad) to detect the spot signals. Two biological replicates of histone peptide array assays were performed for each protein.

The chemiluminescence signal intensities of each spot on histone peptide array slides were quantified using The Array Analyze Software available from the manufacturer (Active Motif) and were assigned the values ranging from 0 (minimum, no signal) to 1 (maximum, strongest signal). The strongest signal for each array was observed at spot “P21” where the myc-tag peptide is spotted given that an anti-myc antibody was used to detect Myc-MBD9 binding. Since MBD9 is not expected to interact with the H2B peptide (spot “P4” on the array) due to differences in the N-terminal amino acid sequences between the human H2B and Arabidopsis H2B [80], the average signal intensity of the “P4” spot was subtracted from the average intensity values for each spot for each array. The average background intensities of the “P4” spots from two arrays (S12A and S12B Fig) were 0.1145 and 0.2605, respectively. After the background subtraction, only the normalized average spot intensities with values higher than 0.3 were presented in Fig 5, along with the spot intensities for H3K4me3 and H3K27me3.

### Purification of protein complexes containing the ARP6-TAP-tag fusion protein

The ARP6-TAP-containing protein complex was purified as described in [45], with the following modifications: 1) instead of using a kitchen blender in a stainless steel wine cooler, 50 grams of frozen seedlings were ground with a mortar and pestle in liquid nitrogen, and 2) all washing steps of the IgG-Sepharose and streptavidin-Sepharose Poly-Prep columns were performed using a peristaltic pump at a flow rate of 1 ml/min at 4°C.

### Mass spectrometry (MS) analysis of proteins co-purified with the ARP6-TAP fusion protein

On-bead digestion of the TAP-purified proteins was performed as previously reported [81, 82]. Residual wash buffer was removed and 200 μl of 50 mM NH_4_HCO_3_ was added to each sample. Samples were reduced with 1 mM dithiothreitol for 30 minutes and alkylated with 5mM iodoacetamide in the dark for an additional 30 minutes. Both steps were performed at room temperature. Digestion was started with the addition of 1 μg of lysyl endopeptidase (Wako) for 2 hours and further digested overnight with 1:50 (w/w) trypsin (Promega) at room temperature. Resulting peptides were acidified with 25 μl of 10% formic acid (FA) and 1% trifluoroacetic acid (TFA), desalted with a Sep-Pak C18 column (Waters), and dried under vacuum.

Liquid chromatography coupled to tandem mass spectrometry (LC-MS/MS) on an Orbitrap Fusion mass spectrometer (ThermoFisher Scientific, San Jose, CA) was performed at the Emory Integrated Proteomics Core (EIPC) [81, 82]. The dried samples were resuspended in 10 μl of loading buffer (0.1% formic acid, 0.03% trifluoroacetic acid, 1% acetonitrile). Peptide mixtures (2 μl) were loaded onto a 25 cm x 75 μm internal diameter fused silica column (New Objective, Woburn, MA) self-packed with 1.9 μm C18 resin (Dr. Maisch, Germany). Separation was carried out over a 140-minute gradient by a Dionex Ultimate 3000 RSLCnano at a flow rate of 300 nl/min. The gradient ranged from 3% to 99% buffer B (buffer A: 0.1% formic acid in water, buffer B: 0.1% formic in ACN). The spectrometer was operated in top speed mode with 3 second cycles. Full MS scans were collected in profile mode at 120,000 resolution at m/z 200 with an automatic gain control (AGC) of 200,000 and a maximum ion injection time of 50 ms. The full mass range was set from 400–1600 m/z. Tandem MS/MS scans were collected in the ion trap after higher-energy collisional dissociation (HCD). The precursor ions were isolated with a 0.7 m/z window and fragmented with 32% collision energy. The product ions were collected with the AGC set for 10,000 and the maximum injection time set to 35 ms. Previously sequenced precursor ions within +/- 10 ppm were excluded from sequencing for 20s using the dynamic exclusion parameters and only precursors with charge states between 2+ and 6+ were allowed.

All raw data files were processed using the Proteome Discoverer 2.1 data analysis suite (Thermo Scientific, San Jose, CA). The database was downloaded from Uniprot and consists of 33,388 *Arabidopsis thaliana* target sequences. An additional sequence was added for the TAP-tagged bait protein. Peptide matches were restricted to fully tryptic cleavage and precursor mass tolerances of +/- 20 ppm and product mass tolerances of +/- 0.6 Daltons. Dynamic modifications were set for methionine oxidation (+15.99492 Da) and protein N-terminal acetylation (+42.03670). A maximum of 3 dynamic modifications were allowed per peptide and a static modification of +57.021465 Da was set for carbamidomethyl cysteine. The Percolator node within Proteome Discoverer was used to filter the peptide spectral match (PSM) false discovery rate to 1%.

### Chromatin Immunoprecipitation (ChIP) with H2A.Z and H4Ac antibodies

For each sample, 1.5 grams of 6-days-old seedlings (with roots removed) were used for ChIP-seq and ChIP-qPCR experiments. The ChIP-seq experiments were performed in biological triplicates on WT, *arp6-1*, and *mbd9-1* seedling tissues as described previously [83], with the following modifications: 1) after the centrifugation of the nuclei in extraction buffer 3, the pellets were resuspended in 210 μl of nuclei lysis buffer, 2) after aliquoting out 10 μl for input, the fragmented chromatin from each sample was split into two equal volumes (100 μl each) and diluted with 1ml of ChIP-dilution buffer, and 3) one diluted half was incubated with 1.5 μg of the affinity-purified polyclonal H2A.Z antibody [41] while the other half was incubated with 5 μl of H4Ac polyclonal antibody serum (Millipore cat.# 06–866). The ChIP-qPCR experiments were performed in duplicates on WT, *arp6-1*, *mbd9-1*, *mbd9-2*, *mbd9-3*, and *arp6-1;mbd9-1* seedling tissues as described previously [83], with the following modifications: 1) after the centrifugation of the nuclei in extraction buffer 3, the pellets were resuspended in 105 μl of nuclei lysis buffer, and 2) after sonication using a Diagenode Bioruptor, 5 μl of the fragmented chromatin was used for input, while 100 μl was diluted with 1 ml of the ChIP dilution buffer. The whole solution was then used for incubation with 1.5 μg of the affinity-purified polyclonal H2A.Z antibody. The ChIP and input DNA samples from the ChIP-qPCR experiment were analyzed by real-time PCR using the *ACT2* (*At3g18780*) 3’ untranslated region sequence as the endogenous control, and with primers that span the genomic regions of *FLC (At5g10140)*, *ASK11* (*At4g34210)*, and *At4 (At5g03545)* genes (S4 Fig), and of *AT2G34202*, *AT1G62760*, *AT5G28410*, *AT4G15960*, *AT1G76740*, and *AT3G21240* genes (S5 Fig). The sequences of the primers used in S6 Fig were previously described [41, 55], while the sequences of the primers used in S8 Fig are listed in S3 Table.

### ChIP-seq library preparation, sequencing, and data analysis

Libraries were prepared starting with 500 pg of ChIP or input DNA samples using the Swift Accel-NGS 2S Plus DNA library kit (Swift Biosciences) according to the manufacturer’s instructions. All libraries were pooled and sequenced using single-end 50 nt reads on an Illumina NextSeq 500 instrument. Reads were mapped to the *Arabidopsis thaliana* genome (TAIR10) using the Bowtie2 package [84]. Quality filtering and sorting of the mapped reads, as well as removal of the reads that mapped to the organellar genomes, was done as previously described [85] using Samtools 1.3.1 [86]. The filtered and sorted BAM files were converted to bigwig format as previously described [85] using deepTools 2.0 software [87]. Correlation plots for the different H2A.Z or H4Ac samples were computed and plotted using the “*multiBamSummary*” and “*plotCorrelation*” functions in the deepTools package.

For visualization, for a given antibody, BAM files of each genotype were scaled to the same number of reads. This was done using the “samtools view -c” and “samtools view -s” commands to count the number of reads in a BAM file and to scale down the global read amounts in a BAM file, respectively. Three scaled, replicate BAM files of each genotype for H2A.Z were combined and converted to a single bigwig file for each genotype. The same was done for each genotype of H4Ac. Average plots and Heatmaps displaying ChIP-seq data were generated using the SeqPlots app [88].

### ChIP-seq peak calling

Peak calling on ChIP-seq data was done by employing the “*Findpeaks*” function of the HOMER package [89] using the input ChIP-seq files as reference and the “*-region*” option to identify sites of read density enrichment. Called peaks were processed using Bedtools [90] to identify peaks called in at least one other replicate for a given genotype. This was done by keeping any peaks that overlapped by at least 50% between biological replicates. The retained peaks were concatenated and then merged together if they overlapped by at least 200 base pairs.

### Identification of MBD9-dependent and MBD9-independent H2A.Z sites

The amount of H2A.Z reads in WT, *mbd9-1*, and *arp6-1* plants present in H2A.Z-enriched peaks reproducibly identified in WT plants was quantified using HTSeq’s *htseq-count* script [91]. Three replicates of counted reads for all three genotypes were then processed using DESeq2 [92]. MBD9-dependent and MBD9-independent peaks were determined from the DESeq2 results comparing wild type and *mbd9-1* counted peaks. Peaks that had a log2 fold change of 0.6 or more and an adjusted p-value less than or equal to 0.05 were designated as MBD9-dependent H2A.Z sites. Peaks with an absolute log2 fold change less than 0.25 were designated as MBD9-independent H2A.Z sites.

### Genomic distribution of ChIP-seq peaks

The PAVIS web tool [93] was used to determine the genomic distribution of H2A.Z ChIP-seq peaks. PAVIS annotates each peak to a genomic feature using the center of the peak. The “upstream” regions were defined as 2,000 bp upstream of the annotated transcription start site, and “downstream” regions were defined as 1,000 bp downstream of the transcription end site.

### Identification of the genes nearest to the H2A.Z ChIP-seq peaks

Genes nearest to the MBD9-dependent and MBD9-independent H2A.Z sites were identified using the “*TSS*” function of the PeakAnnotator 1.4 program [94] as previously described [85].

### Gene ontology analysis

Gene ontology (GO) analysis was carried out on gene lists from S2 Table using two different GO web tools: 1) the AgriGO GO Analysis Toolkit, with default parameters [95, 96], and 2) Gene Ontology enrichment analysis [97]. GO terms that had a false discovery rate (FDR) of 0.05 or less were considered significant.

### Violin plots of FPKM values

Publicly available RNA-seq FPKM values for genes nearest to the MBD9-dependent and MBD9-independent H2A.Z sites were plotted using ggplot2 [98]. Unpaired t-tests were used to determine whether FPKM values were significantly different between the two sets of genes, with P values less than or equal to 0.05 considered as statistically significant.

### Statistical analysis of ChIP-qPCR experiments at MBD-dependent and MBD-independent H2A.Z sites

We performed student t-tests to calculate the significance of fold changes in H2A.Z levels at three MBD9-dependent and three MBD9-independent sites between WT and *mbd9-1* plants (S8 Fig). Based on our ChIP-seq results, we only expect a reduction in H2A.Z levels at MBD9-dependent sites in *mbd9-1* plants compared to WT. Therefore, we performed a one-tail t-test to calculate the significance of H2A.Z depletion at MBD9-dependent sites (S8A Fig). At MBD9-independent sites, however, we expect that H2A.Z levels may vary in either direction in *mbd9-1* plants versus WT. Thus, for MBD9-independent sites we performed a two-tail t-test analysis of qPCR results. Additionally, when the two-tailed t-test is performed on MBD9-dependent ChIP-qPCR results two out of three probed sites (*AT2G34202* and *AT1G62760*) still show statistically significant depletion of H2A.Z levels.

### Real-time PCR (qPCR)

Real-time PCR was performed on the Applied Biosystems StepOnePlus real-time PCR system using SYBR Green as a detection reagent. The 2^-ΔΔCt^ method [99] of relative quantification was used to calculate the fold enrichment. The results presented for ChIP-qPCR experiments are averaged relative quantities from two biological replicates ± SD.

### cDNA production and real-time RT-PCR (qRT-PCR)

Total RNA was isolated from 6-day old seedlings (with roots removed) using the RNeasy plant mini kit (Qiagen). 2 μg of total RNA was converted into cDNA with Super-Script III first strand synthesis kit (Invitrogen). The cDNAs were used as templates for real-time PCR and ran on StepOnePlus real-time PCR system (Applied Biosystems) using SYBR Green as a detection reagent. The 2^-ΔΔCt^ method [99] of relative quantification was used to calculate the fold enrichment. The *PP2A* gene (*AT1G13320)* was used as the endogenous control [100]. The primer sequences used to measure relative expression levels of *PIE1*, *ARP6*, *MBD9*, *SWC4*, *SWC6*, *YAF9a*, *HTA8*, *HTA9*, and *HTA11* in WT, *arp6-1*, and *mbd9-1* plants are listed in S3 Table.

### Size-exclusion chromatography (SEC)

SEC was performed on the HiPrep 16/60 Sephacryl S-400 HR column (GE Healthcare) equilibrated with SEC buffer (the same extraction buffer as described in [45], but without NP-40 detergent). A mixture of protein standards ranging from 669 to 44 kDa (GE Healthcare), resuspended in the SEC buffer, were run on the column to produce a calibration curve of molecular weights versus elution volumes. The slope equation of the calibration curve was then used to calculate the molecular weight of the peak ARP6 SEC fractions. Total protein extracts were isolated from 1 gram of the WT and *mbd9-1* seedling tissues (without roots) using the same extraction buffer that was used for the ARP6-TAP-tag protein complex purification [45]. For each run, between 1.8 and 2.0 ml of the protein extract was loaded onto the column and 1 ml fractions were collected. For each sample, two biological replicates of the SEC experiments were performed and gave nearly identical results.

### Publicly available ChIP-seq and RNA-seq data

Raw data from ChIP-seq experiments performed on young WT seedlings using antibodies against H3K4me3 (GSM2544796, [101]), H3K36me3 (GSM2544797, [101]), H3K9me2 (GSM2366607, [102]), H3K9Ac (GSM2388452, [103]), H3K18Ac (GSM2096925, [104]), H3K27Ac (GSM2096920, [104]), H3K27me3 (GSM2498437, [105]), and H2AK121ub (GSM2367138, [105]), were processed and analyzed with the same procedures as for our ChIP-seq data (see above) and used to generate the average plots presented in Fig 4. The FPKM values from two different RNA-seq experiments (GSM2752981 and GSM2367133, respectively, [105, 106]) were used to compare expression levels in WT plants of genes nearest to the MBD9-dependent and MBD9-independent H2A.Z sites.

### Accession numbers

All ChIP-seq data generated in this study have been deposited to the NCBI GEO database under accession number GSE117391.

## Supporting information

S1 Fig Expression profiles of three H2A.Z proteins and specific H2A.Z-H2B pairs in Arabidopsis. Publicly available microarray expression data from various Arabidopsis tissues (x-axis of all diagrams) [48] were used to compare the expression profiles among the three H2A.Z proteins (A), and between the specific pairs of H2A.Z/H2B histones (B-D). The level of expression for each histone is shown on the y-axis as an absolute value on a logarithmic scale. (A) Expression profiles of the three H2A.Z histones. HTA9 (green line) has the highest, relatively steady expression level in tissues, whereas HTA11 (red line) and HTA8 (blue line) show similar trends of variable expression levels across tissues, with HTA8 being expressed at the lowest level. (B) The expression profiles of HTA11 (green line) and HTB2 (red line) are nearly identical. (C) The expression profiles of HTA9 (green line) and HTB4 (red line) are also highly similar to one another. (D) The expression profiles of HTA9 (green line) and HTB9 (red line) also show a similar pattern.
(TIF)

S2 Fig Additional phenotypes of *mbd9-1* in Arabidopsis. (A) Shape of rosette leaves at the time of bolting. The red arrows point to the serrated edges of rosette leaves in *arp6-1* and *mbd9-1* plants, which are not seen in WT. (B) Number of flowers with more than four petals. Both *arp6-1* and *mbd9-1* plants have significantly higher number of flowers with extra petals when compared to WT.
(TIF)

S3 Fig ChIP-seq read alignment, peak reproducibility, and sample variability. (A) Table shows number of reads that aligned, passed quality filtering, and were non-organellar for each sample. Samples that were converted to bigwigs were scaled to the same number of reads, relative to the lowest number of reads present in a sample of a given histone mark. The last four columns of the table indicate the number of peaks called in the non-scaled samples, the number of reads present in the peaks called for that sample, the Fraction of Reads in Peaks (FRIP) score for that sample, and the number of peaks that replicate between a given genotype by at least 50% and with at least 200 bases of overlap. (B-C) Heatmap of the spearman correlation between each scaled H2AZ sample and the input samples (B) or each scaled H4Ac sample and the input samples (C).
(TIF)

S4 Fig Total levels of H2A.Z and histone H4 acetylation vary in *mbd9-*1 and *arp6-1* mutant plants compared to WT. Total proteins were isolated from young leaves using an acid extraction protocol and equal volumes were loaded in each lane. (A) Western blot for H2A.Z (top panel) and H4Ac (middle panel), and Coomassie stained gel (bottom panel) of total protein extracts from WT, *mbd9-1*, and *arp6-1* plants. Quantification of H2A.Z (B) and H4Ac (C) levels in WT, *mbd9-1*, and *arp6-1* plants. The Coomassie stained gel (bottom panel from (A)) was used to normalize the signals from H2A.Z and H4Ac western blots. H2A.Z and H4Ac levels in WT plants were set to 1.
(TIF)

S5 Fig The expression of SWR1 components and *H2A*.*Z* genes in *arp6-1* and *mbd9-1* mutant plants. The graphs depict average gene expression values ± SD (n = 2 biological replicates) normalized to the expression of endogenous control gene *PP2A* (*AT1G13320*, [100]). The expression levels of all genes in WT plants were set to 1. (A) Relative expression of *PIE1*, *ARP6*, and *MBD9* genes in WT, *arp6-1*, and *mbd9-1* plants as assayed by qRT-PCR. The expression of the *ARP6* gene in *arp6-1* and the *MBD9* gene in *mbd9-1* is barely detectable, indicating that *arp6-1* and *mbd9-1* are null for *ARP6* and *MBD9*, respectively. (B) Relative expression analysis of *HTA8*, *HTA9*, and *HTA11* in WT, *arp6-1*, and *mbd9-1* plants as measured by qRT-PCR. (C) Relative expression of *SWC4*, *SWC6*, and *YAF9a* genes in WT, *arp6-1*, and *mbd9-1* plants as assayed by qRT-PCR. The reduced expression of *SWC6* in a*rp6-1* may indicate that the deposition of H2A.Z is required for proper expression of this gene.
(TIF)

S6 Fig MBD9 is required for H2A.Z deposition at *FLC*, *ASK11*, and *At4* genes. (A) Enrichment of H2A.Z at the *FLC* gene in WT, *arp6-1*, *mbd9-1*, *mbd9-2*, and *mbd9-3* plants. The graph depicts average ChIP fold enrichment ± SD (n = 2 biological replicates) of H2A.Z as calculated by real-time PCR. The primers spanning the regions 2 and 9 of the *FLC* gene were previously described [41]. The *FLC* regions 2 and 9 are enriched for H2A.Z in WT plants, as previously shown [41]. The H2A.Z enrichment at *FLC* regions 2 and 9 is reduced at least 2-fold in *mbd9* plants when compared to WT plants. (B) Enrichment of H2A.Z at *ASK11* and *At4* genes in WT, *arp6-1*, *mbd9-1*, *mbd9-2*, and *mbd9-3* plants as measured by ChIP real-time PCR. The graph depicts average ChIP fold enrichment ± SD (n = 2 biological replicates) of H2A.Z. H2A.Z enrichment at these genes in *mbd9* plants is lost when compared to WT plants. Primers used to measure H2A.Z enrichment at these 2 genes were previously described [55].
(TIF)

S7 Fig H4Ac signal at reproducible H4Ac-enriched sites identified in wild type samples. Heatmaps of the 10,987 H4Ac peaks reproducibly identified in WT plants. Plots are centered on each peak and show a 2 kb window around the peak centers. Color key limits are the same for all the samples shown.
(TIF)

S8 Fig MBD9-dependent sites have significantly depleted levels of H2A.Z in *mbd9-1* plants versus WT when compared to MBD9-independent sites. (A) Enrichment of H2A.Z at MBD9-dependent sites near *AT2G34202*, *AT1G62760*, and *AT5G28410* genes in WT and *mbd9-1* plants. The graph depicts average ChIP fold enrichment ± SD (n = 2 biological replicates) of H2A.Z, as calculated by real-time PCR. The P values, shown above each gene, were calculated using unpaired, one-tail t-test, and represent the significance of reduction in H2A.Z levels between WT and *mbd9-1* at that gene. (B) IGV screen shots of H2A.Z (green) and *mbd9-1* (purple) ChIP-seq reads around MBD9-dependent sites (red). Genes near these sites are shown in navy at the top of the IGV screenshot. Each snapshot corresponds to the ChIP-qPCR plot shown above in A, with the specific sites amplified shown in blue. (C) Enrichment of H2A.Z at MBD9-independent sites near *AT4G15960*, *AT1G76740*, and *AT3G21240* genes in WT and *mbd9-1* plants. The graph depicts average ChIP fold enrichment ± SD (n = 2 biological replicates) of H2A.Z, as calculated by real-time PCR. The P values, shown above each gene, were calculated using unpaired, two-tail t-test and represent the significance of differences in H2A.Z levels between WT and *mbd9-1* at that gene. (D) IGV screen shots of H2A.Z (green) and *mbd9-1* (purple) ChIP-seq reads around MBD9-independent sites (black). Genes near these sites are shown in navy at the top of the IGV screenshot. Each snapshot corresponds to the ChIP-qPCR plot shown above in C, with the specific sites amplified shown in blue.
(TIF)

S9 Fig Genomic distribution of 1391 MBD9-dependent H2A.Z sites and 1505 MBD9-independent H2A.Z sites. (A) Genomic distribution of 1391 MBD-dependent (left) and 1505 MBD9-independent H2A.Z sites (right) was determined using the PAVIS web tool. The “upstream” regions were defined as 2,000 bp upstream of the transcription start site, and “downstream” regions were defined as 1,000 bp downstream of the transcription end site. (B) Distribution of MBD9-dependent (red) and MBD9-independent (blue) peak centers relative to the nearest TSS. (C) Metaplots of (left) H2A.Z signal in wild type plants across protein-coding gene (PCG) bodies of genes nearest to MBD9-dependent (red) or MBD9-independent (blue) peaks, (middle) H2A.Z signal in wild type (red), *mbd9-3* (cyan), or *arp6-1* (dark grey) plants across PCGs nearest to the MBD9-dependent peaks, (right) H2A.Z signal in wild type (blue), *mbd9-3* (green), or *arp6-1* (grey) plants across PCGs nearest to the MBD9-independent peaks.
(TIF)

S10 Fig Chromatin features distribution at MBD9-dependent and MBD9-independent sites. (A) Heatmaps of H2A.Z, H4Ac, H3K27me3, H2AK121Ub, H3K9me2, H3K9Ac, H3K18Ac, H3K27Ac, H3K4me3, and H3K36me3 at the 1391 MBD9-dependent (top) and 1505 MBD9-independent (bottom) H2A.Z sites. The heatmaps are centered on each peak and show a 2 kb window around the peak center and are clustered into 4 k-means clusters using all histone marks for the clustering. (B-D) Clusters from A, matched by chromatin profiles, are shown to facilitate the comparison of chromatin states between MBD9-dependent (D) versus MBD9-independent (I) H2A.Z sites. Dotted red boxes highlight differences between the two types of sites that likely drive the average differences seen in Fig 4.
(TIF)

S11 Fig Transcript levels of genes proximal to MBD9-dependent and MBD9-independent H2A.Z sites. Violin plots of FPKM values for genes nearest to MBD9-dependent H2A.Z sites (D1 and D2) and MBD9-independent H2A.Z sites (ND1 and ND2). FPKM values were obtained from two different publically available datasets of RNA-seq in WT plants, GSM2367133 (D1 and ND1) and GSM2752981 (D2 and ND2). The violin plots are partitioned into quartiles by horizontal lines. Unpaired t-tests were performed comparing D1 to ND1 (p value = 0.0672), and D2 to ND2 (p value = 0.7033) with a significance threshold of p < 0.05.
(TIF)

S12 Fig Histone peptide array assay using the full-length MBD9 protein. Two biological replicates of the assay (A and B) were performed. GFP protein was used as a positive control for protein expression (GFP signal was detected by fluorescent microscope, bottom left in panels A and B), and as a negative control for a histone peptide array assay (no signal detected in peptide array assay, bottom right in panels A and B). (A) The expression of the full-length Myc-MBD9 protein (expected size is ~240 kDa) was confirmed by western blotting using anti-myc antibody (top left). Myc-MBD9 was incubated with the peptide array, which was probed with anti-myc antibody (top right) to detect the signal. The expression of a GFP protein was confirmed using fluorescent scope (bottom left) before it was incubated with the peptide array and probed with anti-myc antibody (bottom right). The strongest signals on the arrays (both A and B, marked with arrows) are from the myc-tag positive control peptides that are on the array. Each array contains two identical subarrays–one on the left and one on the right. (B) A second biological replicate of the expressed Myc-MBD9 protein (top left) incubated with the histone peptide array and probed with anti-myc antibody (top right), and the expressed GFP protein (bottom left) incubated with the array and probed with anti-myc antibody (bottom right).
(TIF)

S13 Fig Introduction of *gMBD9* or *C-TAP-ARP6* constructs into *arp6-1;mbd9-1* double mutant plants rescues the additive phenotype. Plants of the indicated genotypes were grown under long-day conditions and were photographed at 4 weeks of age. Transgenic *gMBD9* in *arp6-1;mbd9-1* and *C-TAP-ARP6* in *arp6-1;mbd9-1* plants both rescue the additive *arp6-1;mbd9-1* phenotype and resemble their corresponding single mutant plants.
(TIF)

S14 Fig The level of H2A.Z at *FLC* locus is not further reduced in *arp6-1;mbd9-1* double mutant plants when compared to *arp6-1*. Enrichment of H2A.Z at the *FLC* gene in WT, *arp6-1*, *mbd9-1*, and *arp6-1;mbd9-1* plants. The graph depicts average ChIP fold enrichment ± SD (n = 2 biological replicates) of H2A.Z as calculated by real-time PCR. The *FLC* regions 2 and 9 are enriched for H2A.Z in WT plants while the level of reduction of H2A.Z at these *FLC* regions in *arp6-1;mbd9-1* double mutant plants is almost identical to the level of H2A.Z reduction detected in *arp6*-1 plants.
(TIF)

S1 Table All proteins co-purified with the ARP6-TAP-tag from three independent TAP experiments. (XLSX)

S2 Table DESeq comparison of H2A.Z ChIP-seq read counts in WT and *mbd9* mutants at reproducible H2A.Z-enriched sites in WT and list of nearest genes to the MBD9-dependent and MBD9-independent H2A.Z sites. (XLSX)

S3 Table Sequences of primers used in qPCR and qRT-PCR experiments. (XLSX)
