# Supplementary figures and images for: Methyl-CpG-binding domain 9 (MBD9) is required for H2A.Z incorporation into chromatin at a subset of H2A.Z-enriched regions in the Arabidopsis genome
Source: PLoS Genet. 2019 Aug 5;15(8):e1008326. doi: 10.1371/journal.pgen.1008326 (PMC6695207; doi:10.1371/journal.pgen.1008326)

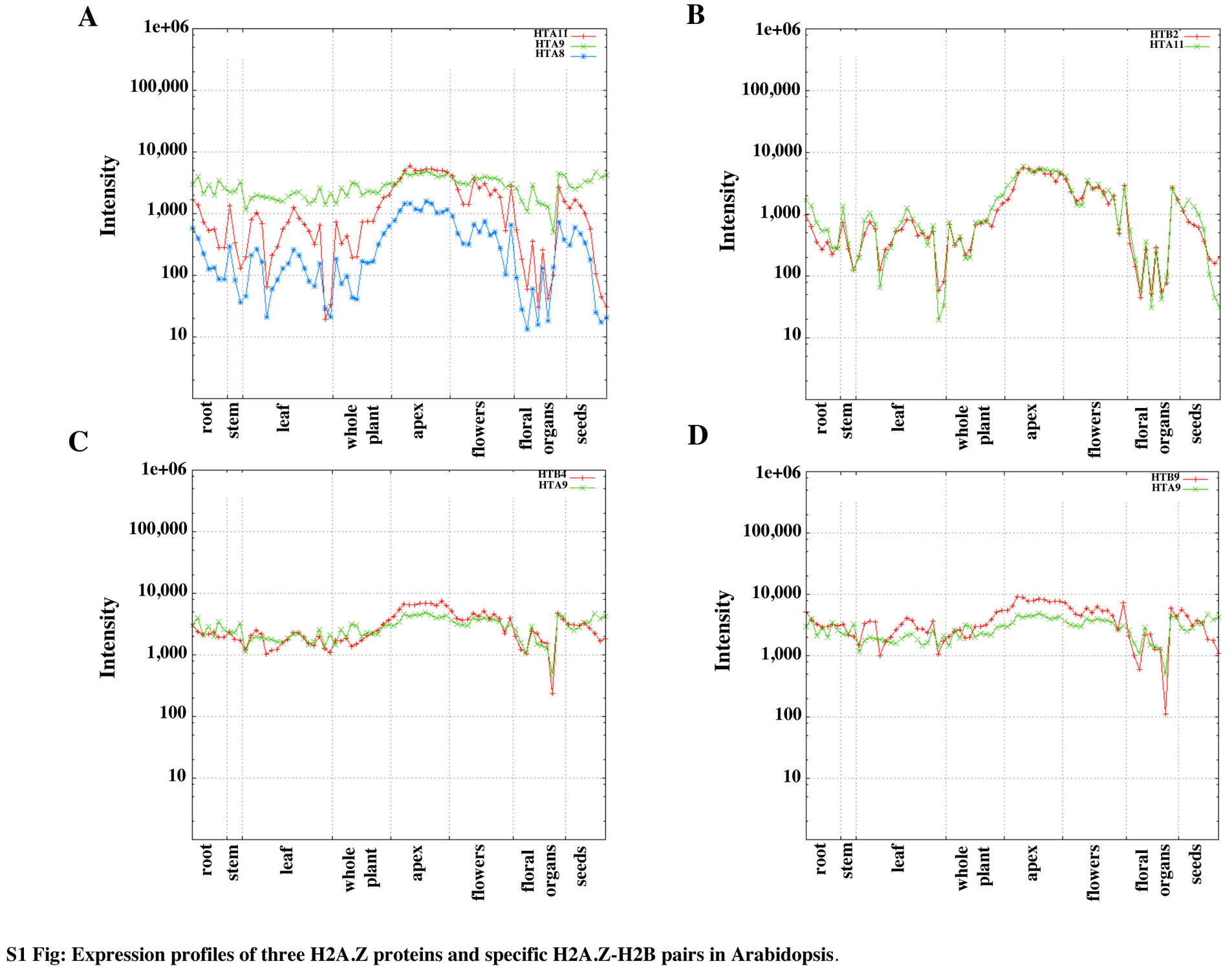

Supplement: S1 Fig — Publicly available microarray expression data from various Arabidopsis tissues (x-axis of all diagrams) [48] were used to compare the expression profiles among the three H2A.Z proteins (A), and between the specific pairs of H2A.Z/H2B histones (B-D). The level of expression for each histone is shown on the y-axis as an absolute value on a logarithmic scale. (A) Expression profiles of the three H2A.Z histones. HTA9 (green line) has the highest, relatively steady expression level in tissues, whereas HTA11 (red line) and HTA8 (blue line) show similar trends of variable expression levels across tissues, with HTA8 being expressed at the lowest level. (B) The expression profiles of HTA11 (green line) and HTB2 (red line) are nearly identical. (C) The expression profiles of HTA9 (green line) and HTB4 (red line) are also highly similar to one another. (D) The expression profiles of HTA9 (green line) and HTB9 (red line) also show a similar pattern. (TIF) [file pgen.1008326.s001.tif]

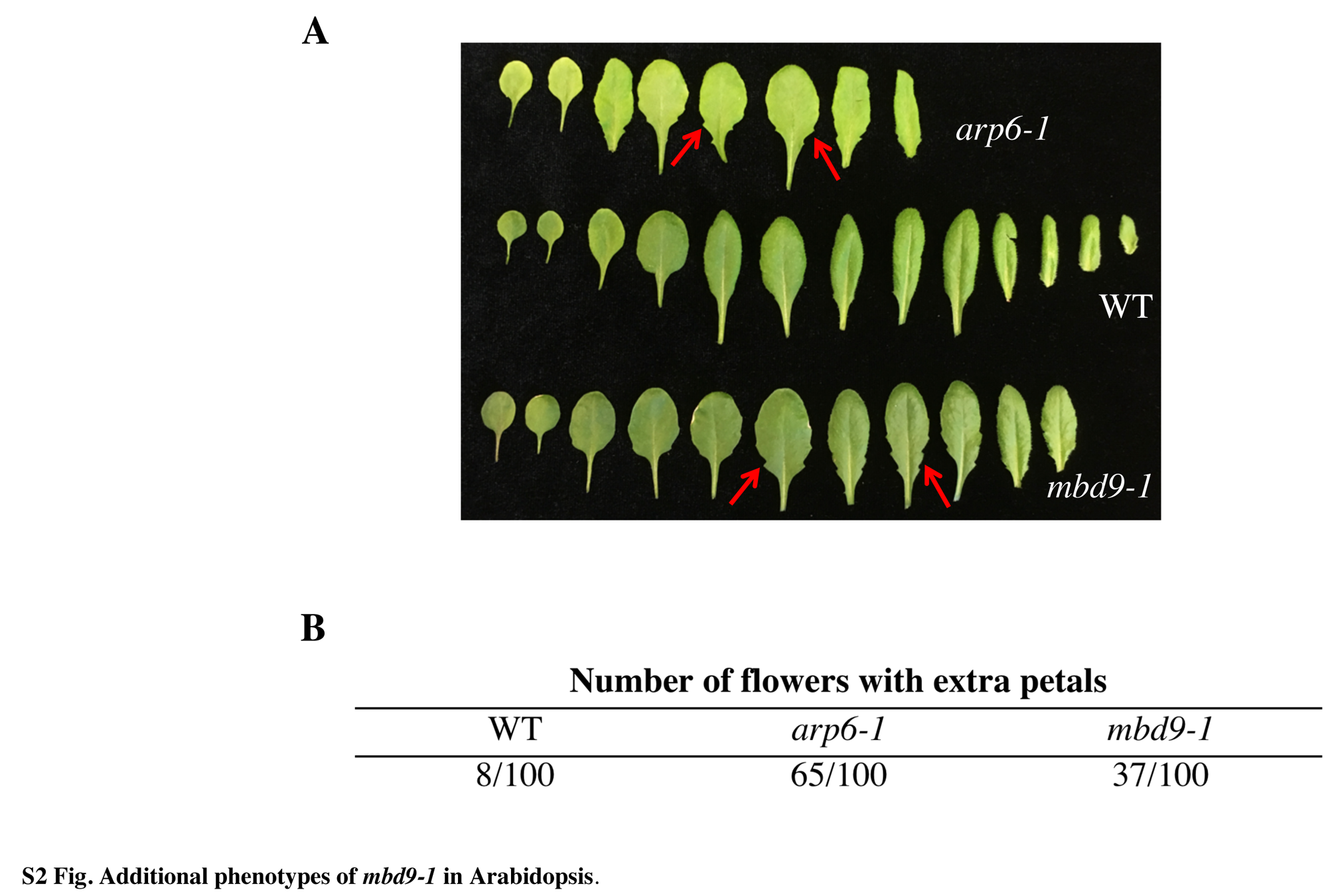

Supplement: S2 Fig — (A) Shape of rosette leaves at the time of bolting. The red arrows point to the serrated edges of rosette leaves in arp6-1 and mbd9-1 plants, which are not seen in WT. (B) Number of flowers with more than four petals. Both arp6-1 and mbd9-1 plants have significantly higher number of flowers with extra petals when compared to WT. (TIF) [file pgen.1008326.s002.tif]

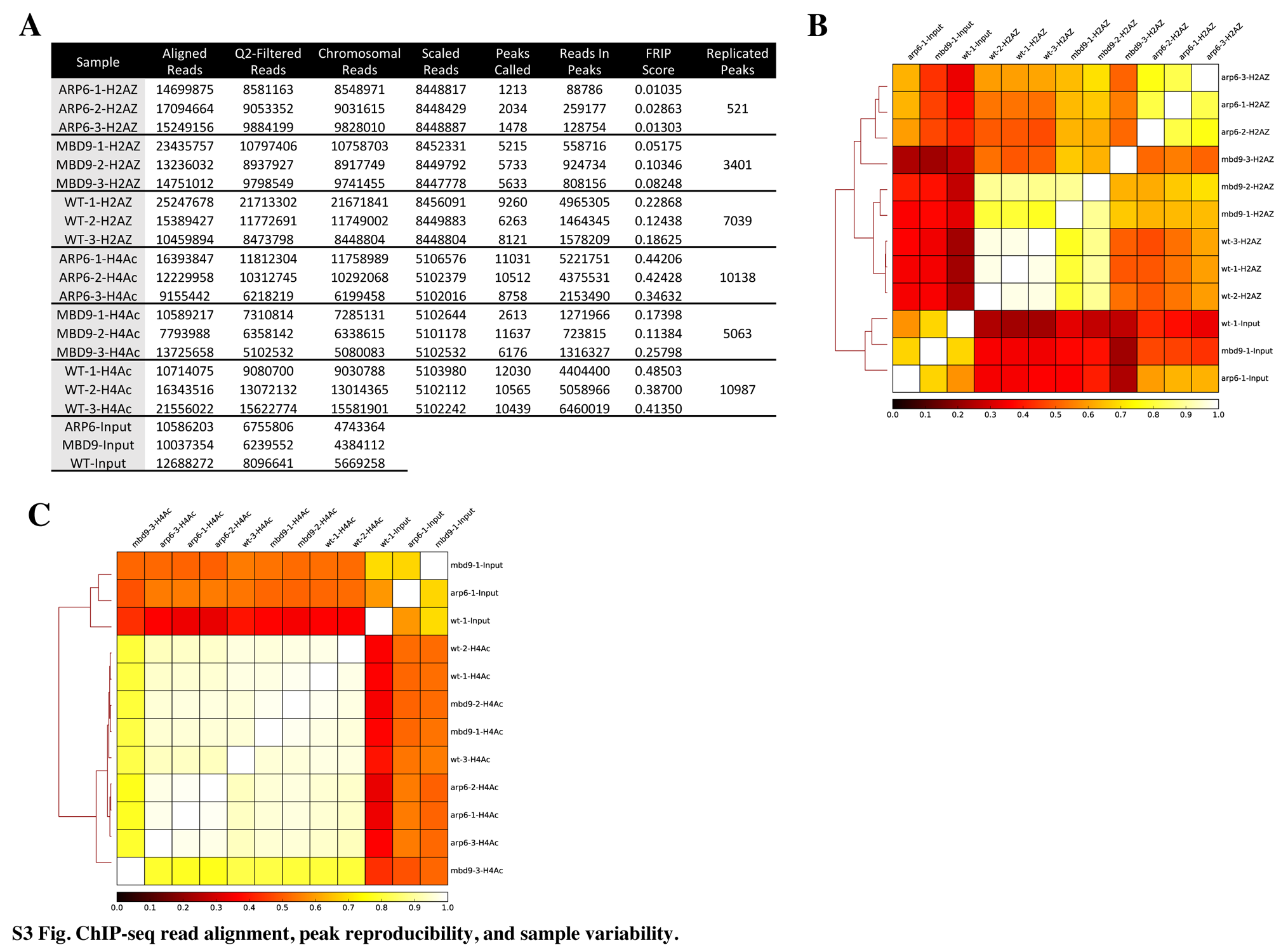

Supplement: S3 Fig — (A) Table shows number of reads that aligned, passed quality filtering, and were non-organellar for each sample. Samples that were converted to bigwigs were scaled to the same number of reads, relative to the lowest number of reads present in a sample of a given histone mark. The last four columns of the table indicate the number of peaks called in the non-scaled samples, the number of reads present in the peaks called for that sample, the Fraction of Reads in Peaks (FRIP) score for that sample, and the number of peaks that replicate between a given genotype by at least 50% and with at least 200 bases of overlap. (B-C) Heatmap of the spearman correlation between each scaled H2AZ sample and the input samples (B) or each scaled H4Ac sample and the input samples (C). (TIF) [file pgen.1008326.s003.tif]

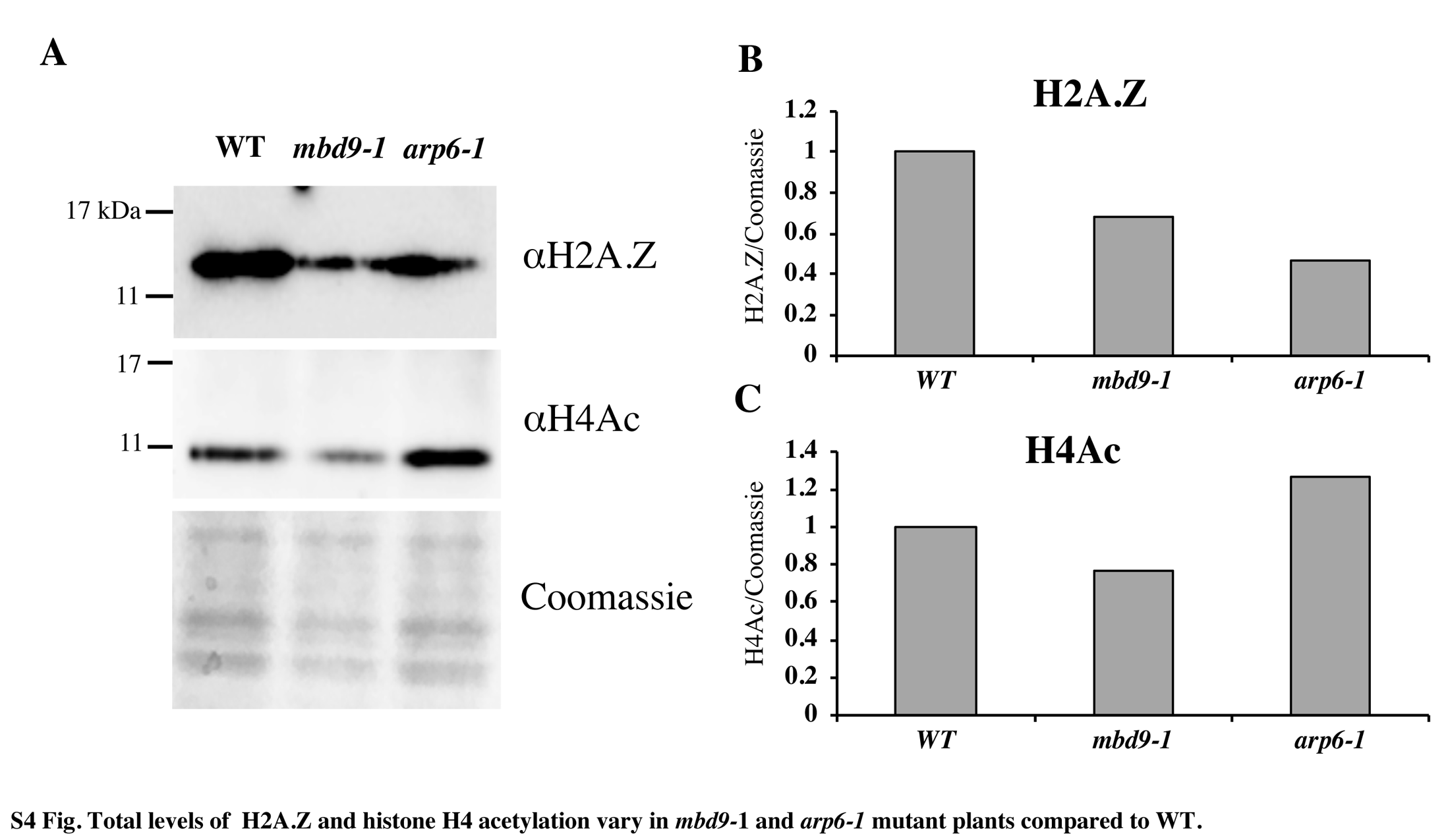

Supplement: S4 Fig — Total proteins were isolated from young leaves using an acid extraction protocol and equal volumes were loaded in each lane. (A) Western blot for H2A.Z (top panel) and H4Ac (middle panel), and Coomassie stained gel (bottom panel) of total protein extracts from WT, mbd9-1, and arp6-1 plants. Quantification of H2A.Z (B) and H4Ac (C) levels in WT, mbd9-1, and arp6-1 plants. The Coomassie stained gel (bottom panel from (A)) was used to normalize the signals from H2A.Z and H4Ac western blots. H2A.Z and H4Ac levels in WT plants were set to 1. (TIF) [file pgen.1008326.s004.tif]

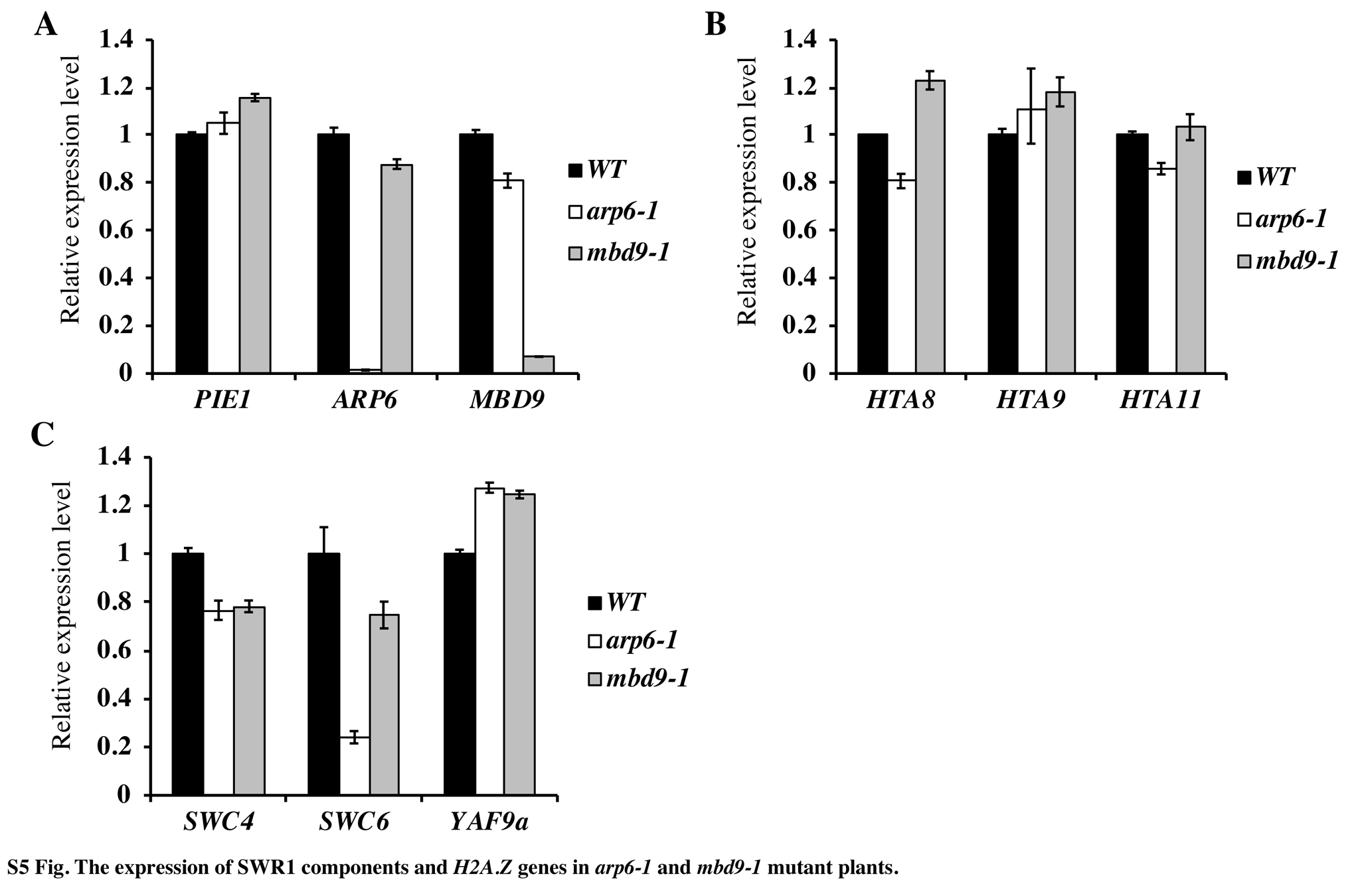

Supplement: S5 Fig — The graphs depict average gene expression values ± SD (n = 2 biological replicates) normalized to the expression of endogenous control gene PP2A (AT1G13320, [100]). The expression levels of all genes in WT plants were set to 1. (A) Relative expression of PIE1, ARP6, and MBD9 genes in WT, arp6-1, and mbd9-1 plants as assayed by qRT-PCR. The expression of the ARP6 gene in arp6-1 and the MBD9 gene in mbd9-1 is barely detectable, indicating that arp6-1 and mbd9-1 are null for ARP6 and MBD9, respectively. (B) Relative expression analysis of HTA8, HTA9, and HTA11 in WT, arp6-1, and mbd9-1 plants as measured by qRT-PCR. (C) Relative expression of SWC4, SWC6, and YAF9a genes in WT, arp6-1, and mbd9-1 plants as assayed by qRT-PCR. The reduced expression of SWC6 in arp6-1 may indicate that the deposition of H2A.Z is required for proper expression of this gene. (TIF) [file pgen.1008326.s005.tif]

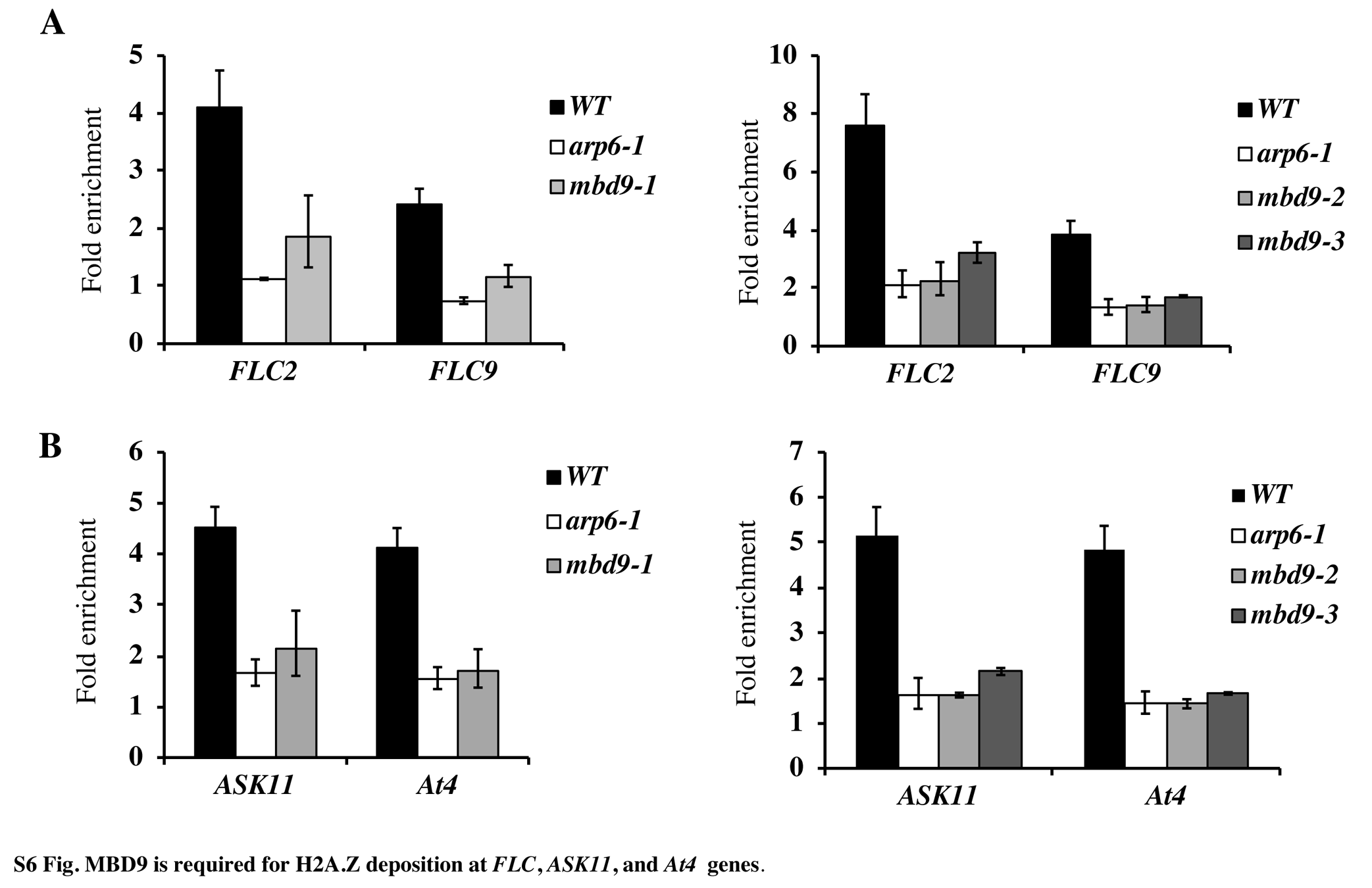

Supplement: S6 Fig — (A) Enrichment of H2A.Z at the FLC gene in WT, arp6-1, mbd9-1, mbd9-2, and mbd9-3 plants. The graph depicts average ChIP fold enrichment ± SD (n = 2 biological replicates) of H2A.Z as calculated by real-time PCR. The primers spanning the regions 2 and 9 of the FLC gene were previously described [41]. The FLC regions 2 and 9 are enriched for H2A.Z in WT plants, as previously shown [41]. The H2A.Z enrichment at FLC regions 2 and 9 is reduced at least 2-fold in mbd9 plants when compared to WT plants. (B) Enrichment of H2A.Z at ASK11 and At4 genes in WT, arp6-1, mbd9-1, mbd9-2, and mbd9-3 plants as measured by ChIP real-time PCR. The graph depicts average ChIP fold enrichment ± SD (n = 2 biological replicates) of H2A.Z. H2A.Z enrichment at these genes in mbd9 plants is lost when compared to WT plants. Primers used to measure H2A.Z enrichment at these 2 genes were previously described [55]. (TIF) [file pgen.1008326.s006.tif]

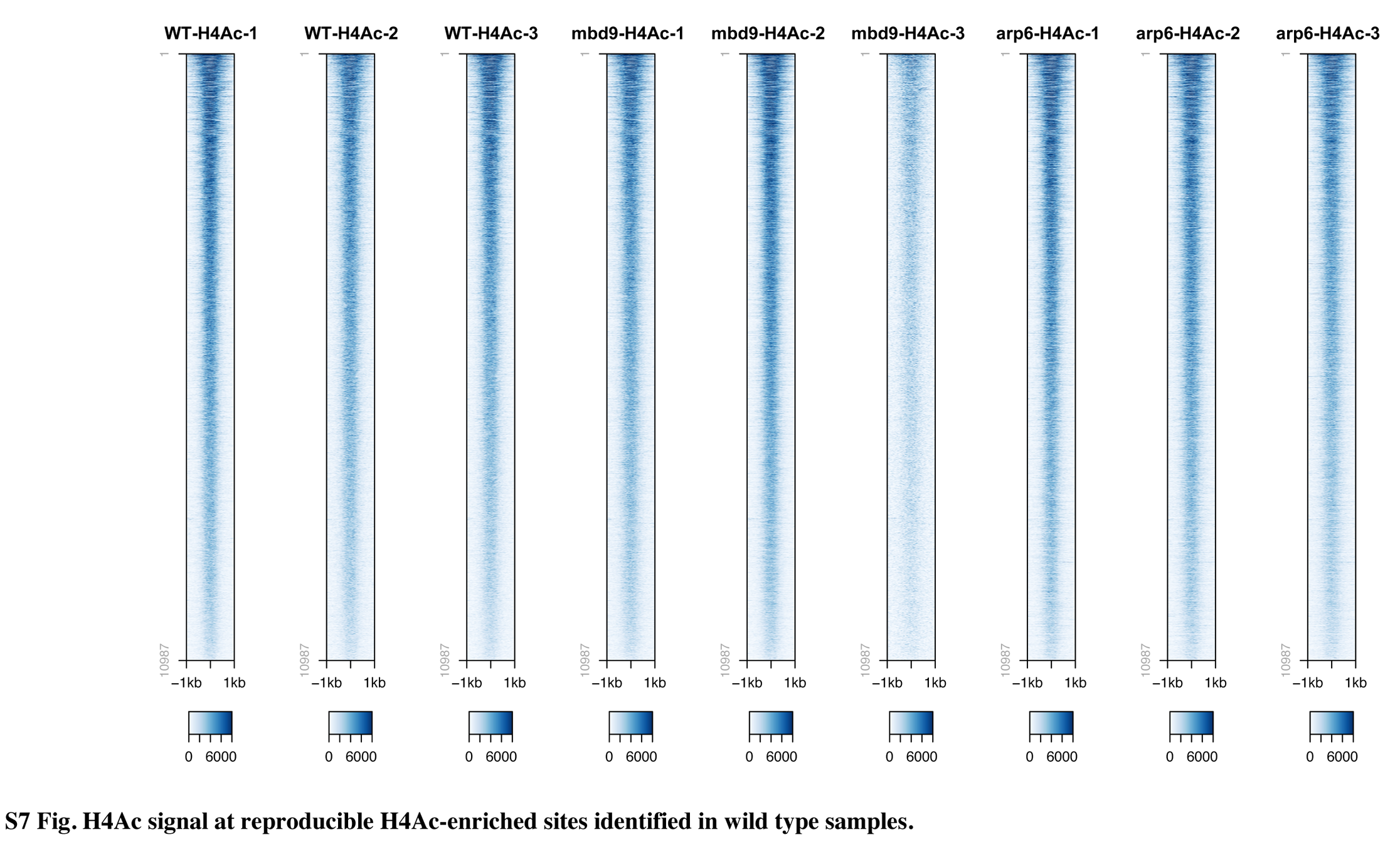

Supplement: S7 Fig — Heatmaps of the 10,987 H4Ac peaks reproducibly identified in WT plants. Plots are centered on each peak and show a 2 kb window around the peak centers. Color key limits are the same for all the samples shown. (TIF) [file pgen.1008326.s007.tif]

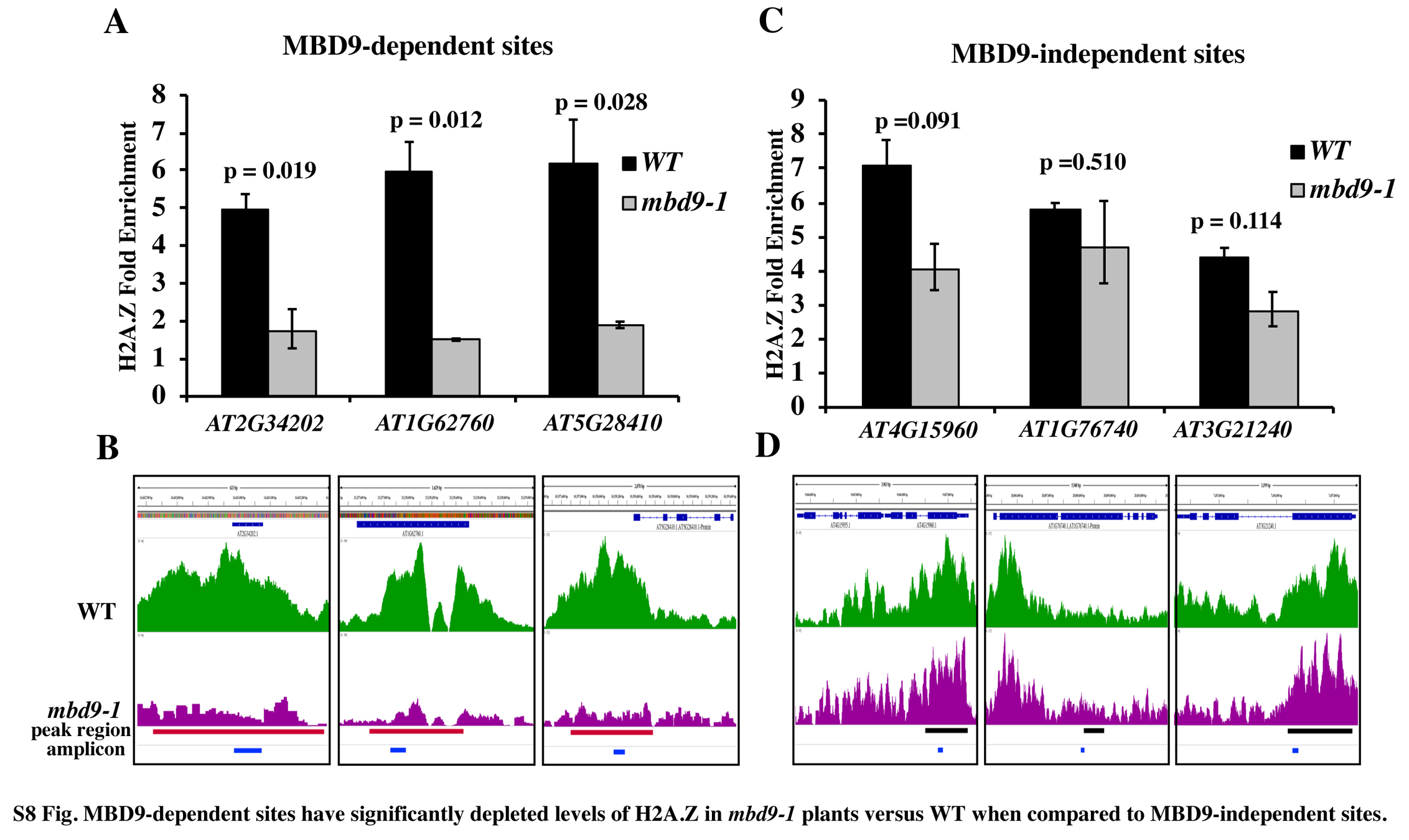

Supplement: S8 Fig — (A) Enrichment of H2A.Z at MBD9-dependent sites near AT2G34202, AT1G62760, and AT5G28410 genes in WT and mbd9-1 plants. The graph depicts average ChIP fold enrichment ± SD (n = 2 biological replicates) of H2A.Z, as calculated by real-time PCR. The P values, shown above each gene, were calculated using unpaired, one-tail t-test, and represent the significance of reduction in H2A.Z levels between WT and mbd9-1 at that gene. (B) IGV screen shots of H2A.Z (green) and mbd9-1 (purple) ChIP-seq reads around MBD9-dependent sites (red). Genes near these sites are shown in navy at the top of the IGV screenshot. Each snapshot corresponds to the ChIP-qPCR plot shown above in A, with the specific sites amplified shown in blue. (C) Enrichment of H2A.Z at MBD9-independent sites near AT4G15960, AT1G76740, and AT3G21240 genes in WT and mbd9-1 plants. The graph depicts average ChIP fold enrichment ± SD (n = 2 biological replicates) of H2A.Z, as calculated by real-time PCR. The P values, shown above each gene, were calculated using unpaired, two-tail t-test and represent the significance of differences in H2A.Z levels between WT and mbd9-1 at that gene. (D) IGV screen shots of H2A.Z (green) and mbd9-1 (purple) ChIP-seq reads around MBD9-independent sites (black). Genes near these sites are shown in navy at the top of the IGV screenshot. Each snapshot corresponds to the ChIP-qPCR plot shown above in C, with the specific sites amplified shown in blue. (TIF) [file pgen.1008326.s008.tif]

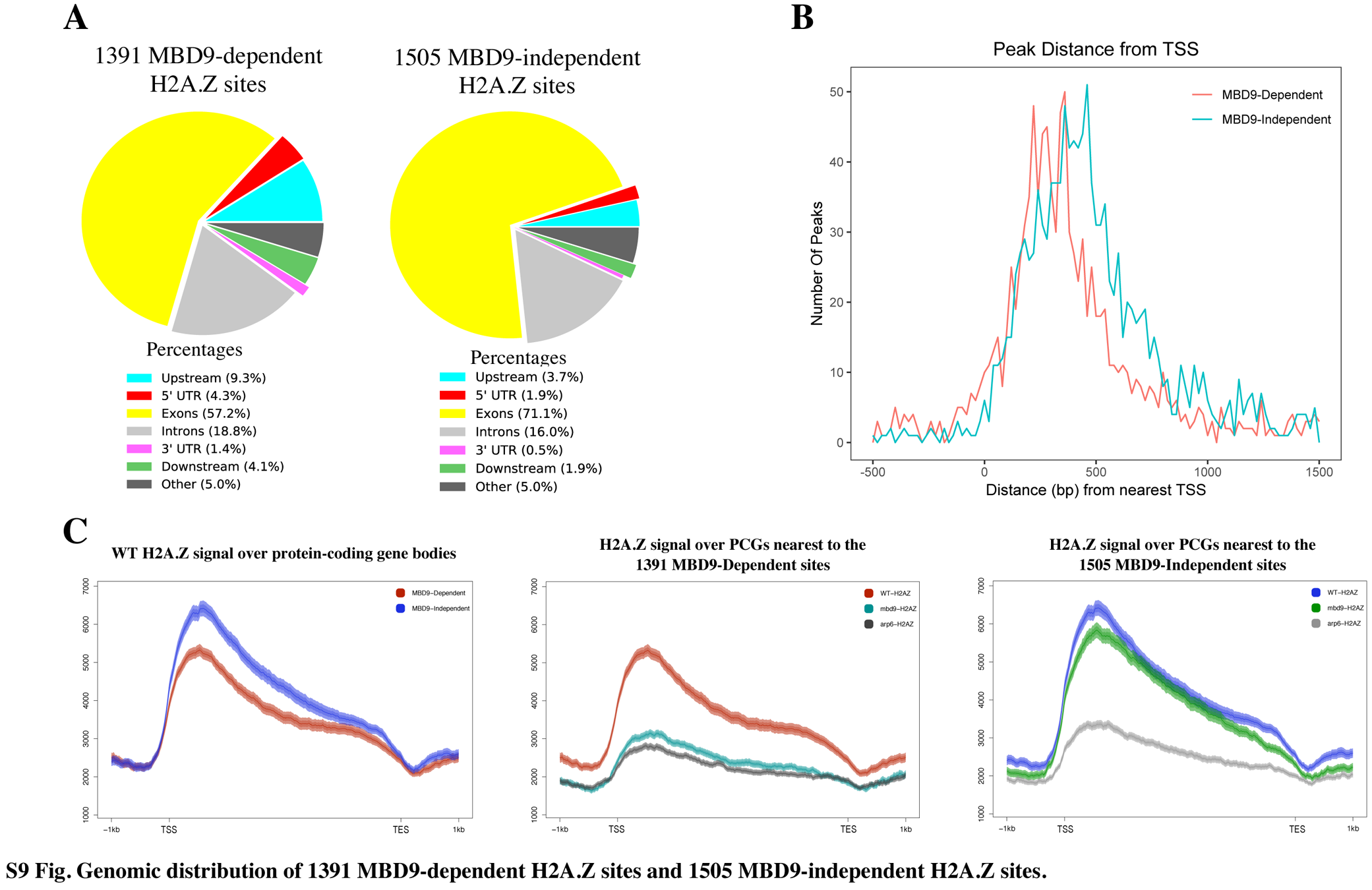

Supplement: S9 Fig — (A) Genomic distribution of 1391 MBD-dependent (left) and 1505 MBD9-independent H2A.Z sites (right) was determined using the PAVIS web tool. The “upstream” regions were defined as 2,000 bp upstream of the transcription start site, and “downstream” regions were defined as 1,000 bp downstream of the transcription end site. (B) Distribution of MBD9-dependent (red) and MBD9-independent (blue) peak centers relative to the nearest TSS. (C) Metaplots of (left) H2A.Z signal in wild type plants across protein-coding gene (PCG) bodies of genes nearest to MBD9-dependent (red) or MBD9-independent (blue) peaks, (middle) H2A.Z signal in wild type (red), mbd9-3 (cyan), or arp6-1 (dark grey) plants across PCGs nearest to the MBD9-dependent peaks, (right) H2A.Z signal in wild type (blue), mbd9-3 (green), or arp6-1 (grey) plants across PCGs nearest to the MBD9-independent peaks. (TIF) [file pgen.1008326.s009.tif]

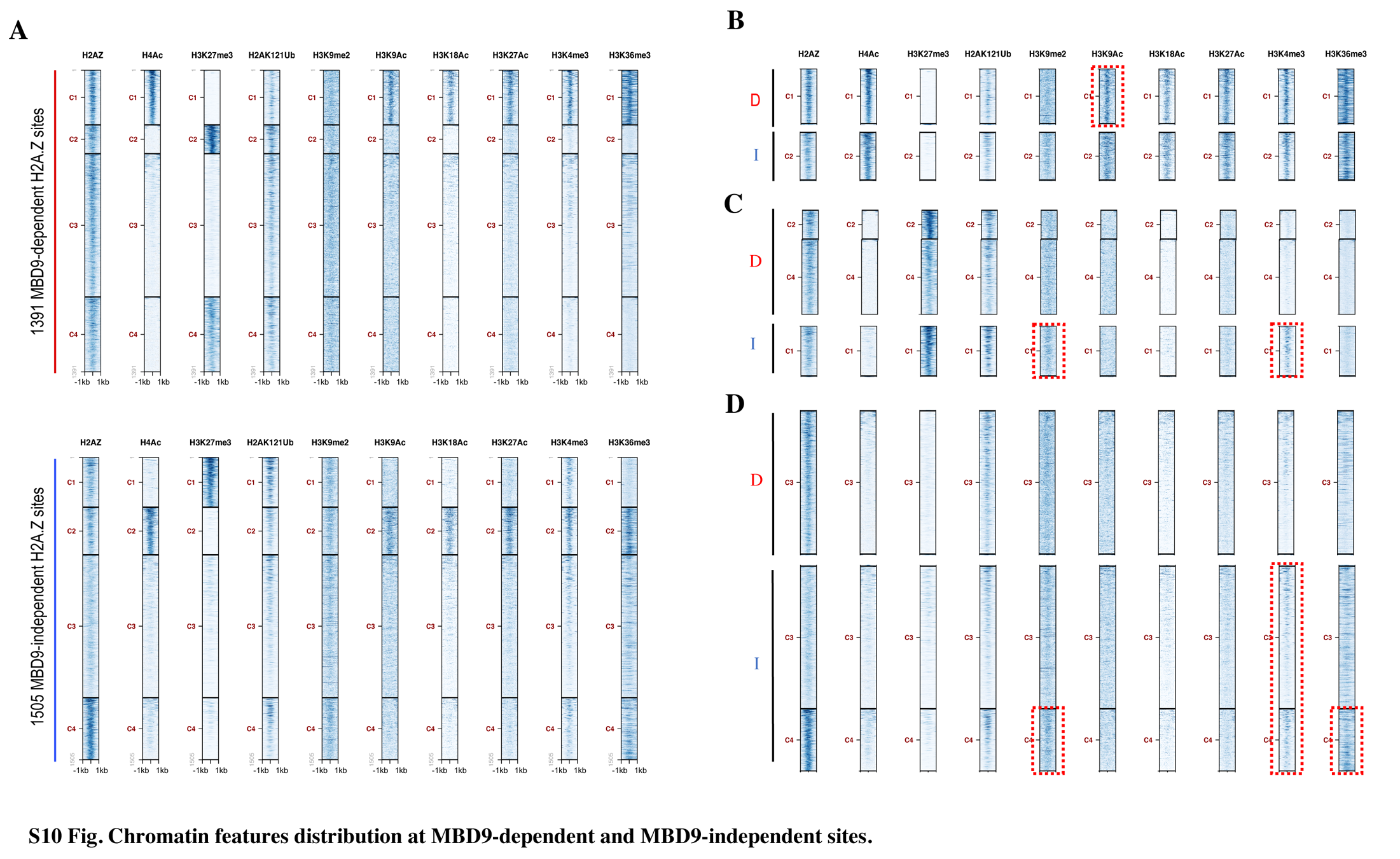

Supplement: S10 Fig — (A) Heatmaps of H2A.Z, H4Ac, H3K27me3, H2AK121Ub, H3K9me2, H3K9Ac, H3K18Ac, H3K27Ac, H3K4me3, and H3K36me3 at the 1391 MBD9-dependent (top) and 1505 MBD9-independent (bottom) H2A.Z sites. The heatmaps are centered on each peak and show a 2 kb window around the peak center and are clustered into 4 k-means clusters using all histone marks for the clustering. (B-D) Clusters from A, matched by chromatin profiles, are shown to facilitate the comparison of chromatin states between MBD9-dependent (D) versus MBD9-independent (I) H2A.Z sites. Dotted red boxes highlight differences between the two types of sites that likely drive the average differences seen in Fig 4. (TIF) [file pgen.1008326.s010.tif]

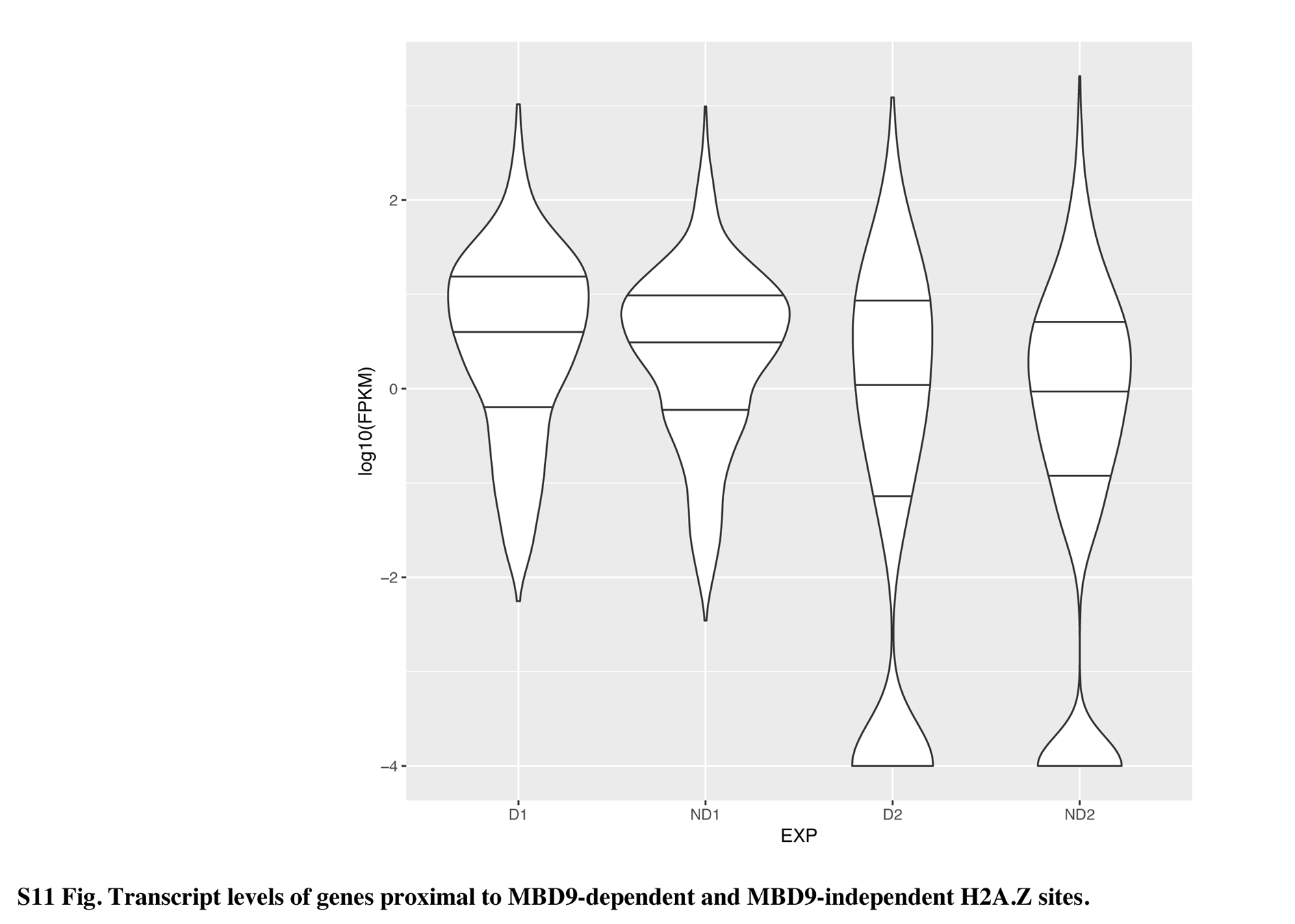

Supplement: S11 Fig — Violin plots of FPKM values for genes nearest to MBD9-dependent H2A.Z sites (D1 and D2) and MBD9-independent H2A.Z sites (ND1 and ND2). FPKM values were obtained from two different publically available datasets of RNA-seq in WT plants, GSM2367133 (D1 and ND1) and GSM2752981 (D2 and ND2). The violin plots are partitioned into quartiles by horizontal lines. Unpaired t-tests were performed comparing D1 to ND1 (p value = 0.0672), and D2 to ND2 (p value = 0.7033) with a significance threshold of p < 0.05. (TIF) [file pgen.1008326.s011.tif]

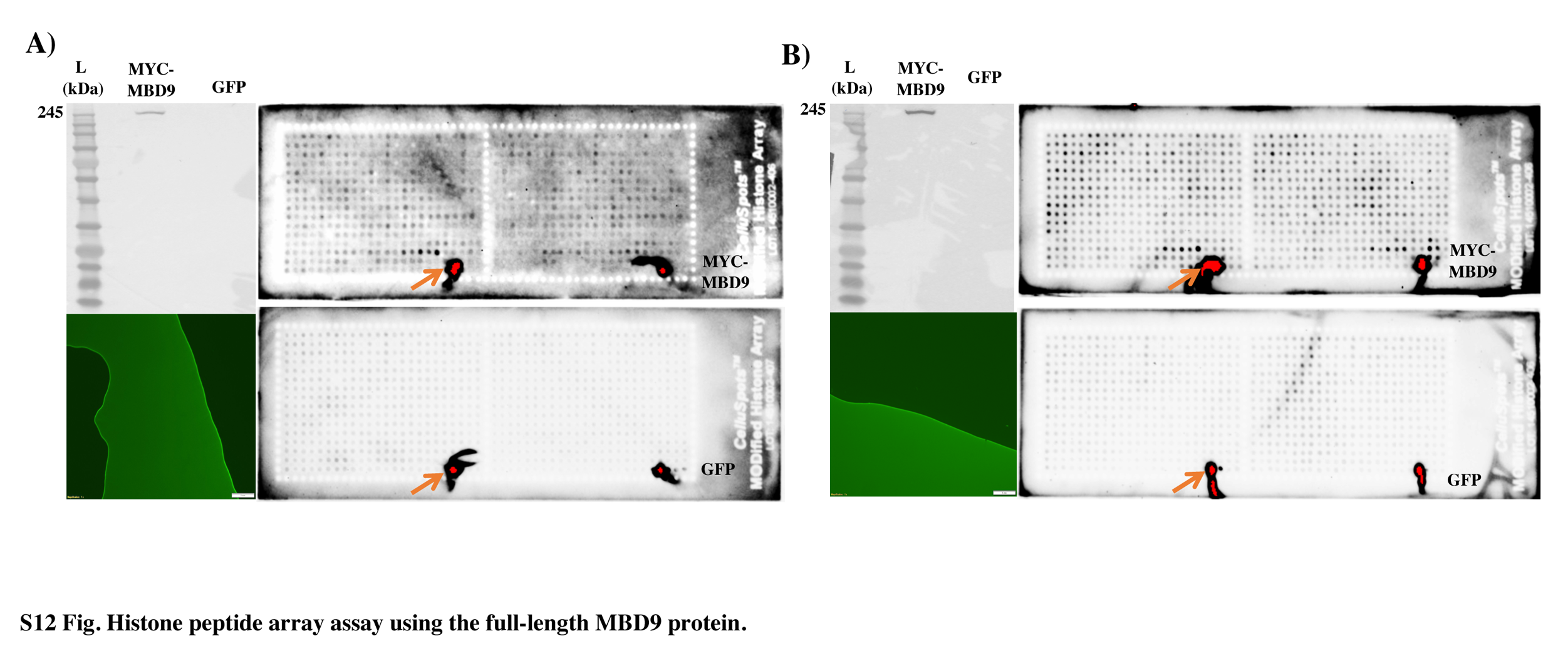

Supplement: S12 Fig — Two biological replicates of the assay (A and B) were performed. GFP protein was used as a positive control for protein expression (GFP signal was detected by fluorescent microscope, bottom left in panels A and B), and as a negative control for a histone peptide array assay (no signal detected in peptide array assay, bottom right in panels A and B). (A) The expression of the full-length Myc-MBD9 protein (expected size is ~240 kDa) was confirmed by western blotting using anti-myc antibody (top left). Myc-MBD9 was incubated with the peptide array, which was probed with anti-myc antibody (top right) to detect the signal. The expression of a GFP protein was confirmed using fluorescent scope (bottom left) before it was incubated with the peptide array and probed with anti-myc antibody (bottom right). The strongest signals on the arrays (both A and B, marked with arrows) are from the myc-tag positive control peptides that are on the array. Each array contains two identical subarrays–one on the left and one on the right. (B) A second biological replicate of the expressed Myc-MBD9 protein (top left) incubated with the histone peptide array and probed with anti-myc antibody (top right), and the expressed GFP protein (bottom left) incubated with the array and probed with anti-myc antibody (bottom right). (TIF) [file pgen.1008326.s012.tif]

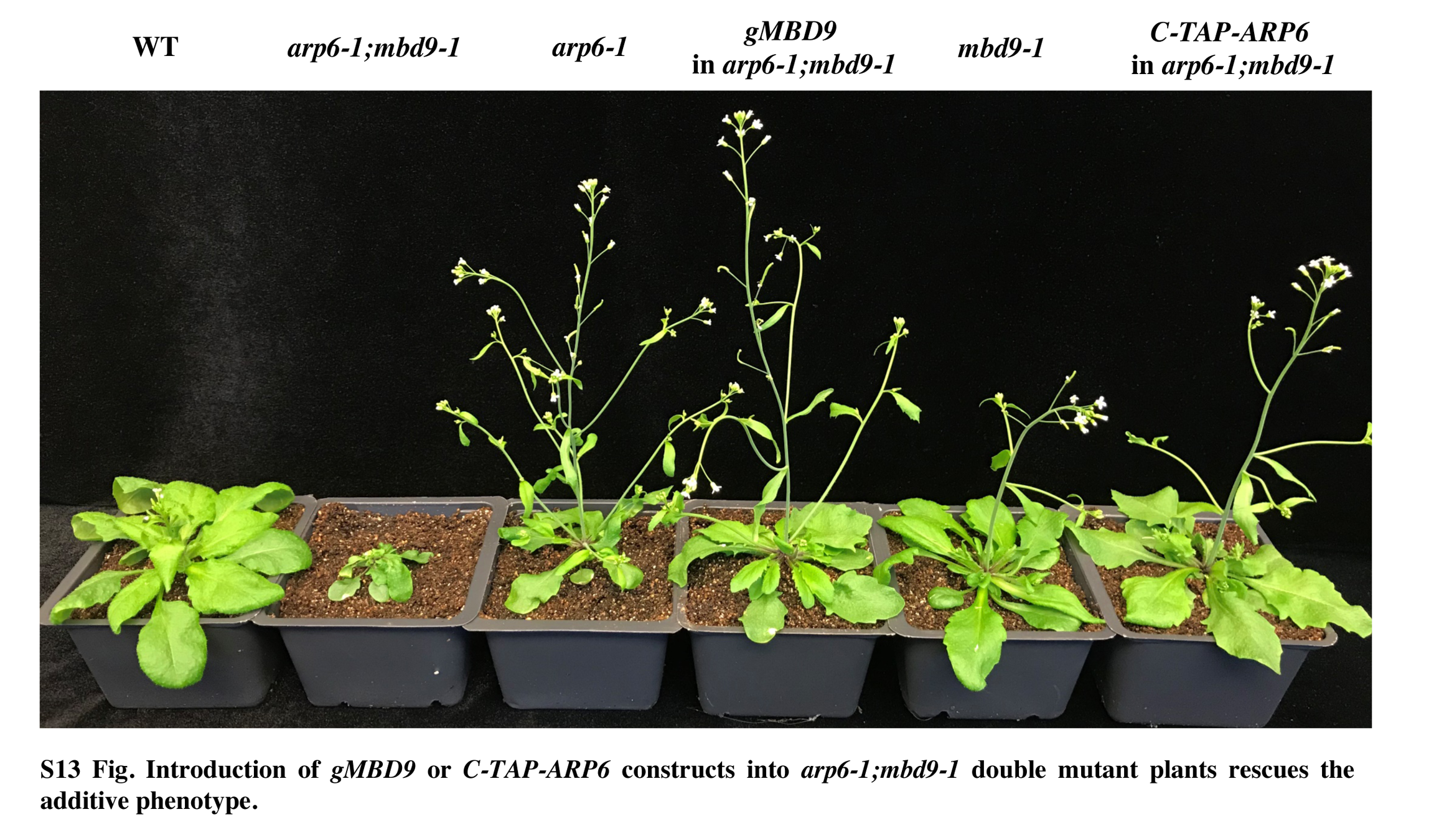

Supplement: S13 Fig — Plants of the indicated genotypes were grown under long-day conditions and were photographed at 4 weeks of age. Transgenic gMBD9 in arp6-1;mbd9-1 and C-TAP-ARP6 in arp6-1;mbd9-1 plants both rescue the additive arp6-1;mbd9-1 phenotype and resemble their corresponding single mutant plants. (TIF) [file pgen.1008326.s013.tif]

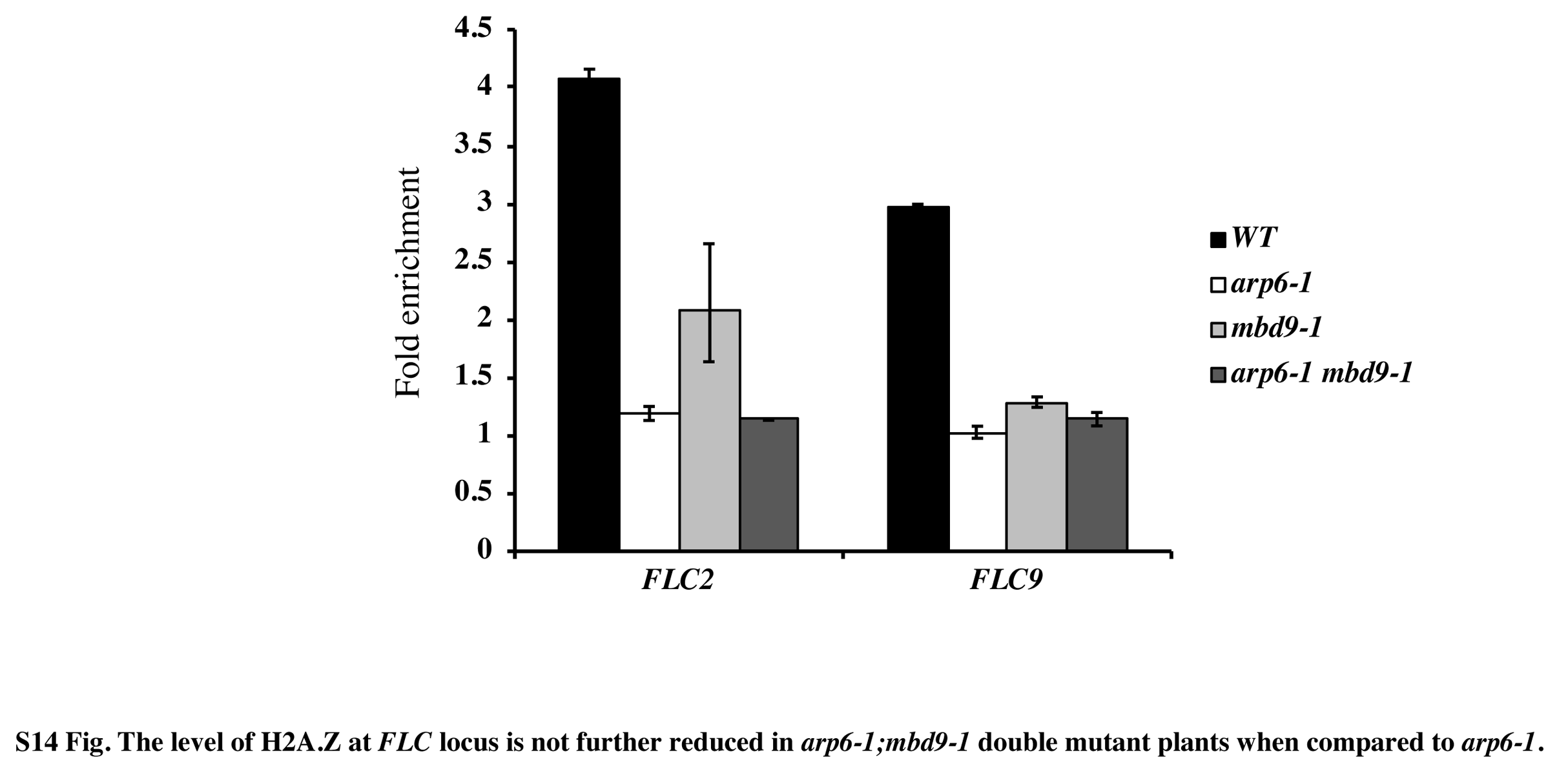

Supplement: S14 Fig — Enrichment of H2A.Z at the FLC gene in WT, arp6-1, mbd9-1, and arp6-1;mbd9-1 plants. The graph depicts average ChIP fold enrichment ± SD (n = 2 biological replicates) of H2A.Z as calculated by real-time PCR. The FLC regions 2 and 9 are enriched for H2A.Z in WT plants while the level of reduction of H2A.Z at these FLC regions in arp6-1;mbd9-1 double mutant plants is almost identical to the level of H2A.Z reduction detected in arp6-1 plants. (TIF) [file pgen.1008326.s014.tif]
